# Genome-wide association study of individual differences of human lymphocyte profiles using large-scale cytometry data

**DOI:** 10.1038/s10038-020-00874-x

**Published:** 2020-11-23

**Authors:** Daigo Okada, Naotoshi Nakamura, Kazuya Setoh, Takahisa Kawaguchi, Koichiro Higasa, Yasuharu Tabara, Fumihiko Matsuda, Ryo Yamada

**Affiliations:** 1 Department of Statistical Genetics, Center for Genomic Medicine, Graduate School of Medicine, Kyoto University, Kyoto, Japan; 2 Department of Human Disease Genomics, Center for Genomic Medicine, Graduate School of Medicine, Kyoto University, Kyoto, Japan; 3 Department of Genome Analysis, Institute of Biomedical Science, Kansai Medical University, Hirakata, Japan

**Keywords:** Genome-wide association studies, Data mining

## Abstract

Human immune systems are very complex, and the basis for individual differences in immune phenotypes is largely unclear. One reason is that the phenotype of the immune system is so complex that it is very difficult to describe its features and quantify differences between samples. To identify the genetic factors that cause individual differences in whole lymphocyte profiles and their changes after vaccination without having to rely on biological assumptions, we performed a genome-wide association study (GWAS), using cytometry data. Here, we applied computational analysis to the cytometry data of 301 people before receiving an influenza vaccine, and 1, 7, and 90 days after the vaccination to extract the feature statistics of the lymphocyte profiles in a nonparametric and data-driven manner. We analyzed two types of cytometry data: measurements of six markers for B cell classification and seven markers for T cell classification. The coordinate values calculated by this method can be treated as feature statistics of the lymphocyte profile. Next, we examined the genetic basis of individual differences in human immune phenotypes with a GWAS for the feature statistics, and we newly identified seven significant and 36 suggestive single-nucleotide polymorphisms associated with the individual differences in lymphocyte profiles and their change after vaccination. This study provides a new workflow for performing combined analyses of cytometry data and other types of genomics data.

## Introduction

The human immune system is highly complex [1]. It is still unclear what individual differences exist in the phenotype of a healthy person’s immune system. Also, it is not clear how the immune system phenotype changes with the immune response. One reason is that the phenotype of the immune system is so complex that it is very difficult to describe its features and quantify differences between samples.

To investigate complex biological phenomena, such as the immune response to vaccination, genome-wide association studies (GWASs) are a powerful approach. They can detect the single-nucleotide polymorphisms (SNPs) that are associated with complex traits [2]. For the immune response to vaccination, several GWAS analyses have been conducted, and in these analyses, the blood cytokine measurement or titer [3, 4] has been used to represent the immune response. These previous studies have successfully detected genetic variants associated with the immune response to vaccination. However, the immunophenotype is very complex and difficult to comprehensively characterize by using the concentrations of single blood metabolites.

The immunophenotype is not only a complex trait but is also strongly characterized by the lymphocyte profile, as measured by cytometry data [5]. Recently, large-scale flow cytometry data analyses of the immune response to vaccination have revealed differentially expressed genes before and after vaccination, in addition to crucial subsets of the immune response [6–8]. However, these studies have not focused on individual differences, but rather the general mechanism of the immune response to vaccination, and then only on specific lymphocyte subsets based on the previous biological knowledge.

In the field of computational biology, several methods have been proposed to examine the differences of cell population profiles among multiple cytometry samples in a data-driven and nonparametric manner [9–11]. In these approaches, cytometry data are considered as a sample from an unknown multidimensional probability distribution. These methods quantify dissimilarities between probability distributions based on information theory, apply a multidimensional scaling (MDS) method to the distance matrix, and then embed them in a low-dimensional space. The obtained coordinate values can be treated as feature statistics of the cell population profile, which enables sample differences to be visualized in low-dimensional space.

In this study, we used a computational method for large-scale cytometry data and embedded the lymphocyte profiles into low-dimensional spaces based on the dissimilarities among samples. The coordinate values calculated by this method can be treated as feature statistics of the lymphocyte profile. To conduct the following analyses, the extraction of some feature statistics from cytometry data is a necessary step, which enables us to examine the correlation with SNP genotype without needing biological assumptions. We identified new SNPs related to the individual differences in lymphocyte profiles and their changes after vaccination via a GWAS for these feature statistics. Our results provide novel insights into the genetics of individual differences of the immune response.

## Materials and methods

### Flow cytometry and SNP genotype data

In this study, we used data that we obtained from a related project with the Nagahama Cohort Study [12]. This project profiled 301 healthy people (103 men and 198 women) aged between 32 and 66. The participants had received an injection of trivalent inactivated influenza vaccine that contained three types of HA antigens from A/California/7/2009 (H1N1) pdm09, A/Victoria/210/2009 (H3N2), and B/Brisbane/60/2008. Peripheral blood was collected at four time points, before influenza vaccine (Day 0) and 1 day (Day 1), 7 days (Day 7), and 90 days (Day 90) after vaccination. Although FACS data were taken at all four time points, a total of 1173 samples were used because of partial loss. Two types of FACS data (B cell FACS and T cell FACS) were obtained for each person at each time point. In B cell FACS, a set of six cell surface markers (CD19, IgM, IgD, CD21, CD27, and CD138) for B cell classification were measured. These markers can be used to identify plasma cells, immature B cells, naive B cells, non-switched memory B cells, class-switched memory B cells, and double-negative memory B cells with conventional gating methods [13–15]. For T cell FACS, a set of seven cell surface markers (CD3, CD4, CD8, CD45RA, CD45RO, CD25, and CCR7) for T cell classification were measured. CD4 and CD8 can be used to identify helper T cells, killer T cells, and double-negative T cells. CD45RA, CD45RO, and CD25 can be used to identify naive T cells, memory T cells, and effector T cells [16–23]. CCR7 is a marker for identifying exhausted T cells (CCR7 negative) from naive T cells (CCR7 positive), and for classifying memory T cells into central memory T cells and effector memory T cells. These markers may not be sufficient to accurately classify all B cell and T cell subsets. For example, CD25 is known to be a marker of regulatory T cells, as well as in the T cell subset described above [24], and plasma cells that do not express CD138 are also present [25]. We selected these marker sets to capture the information of as many lymphocyte subsets as possible with a limited number of markers, rather than for quantification and classification of each subset.

All FACS data were preprocessed with compensation, normalization by inverse hyperbolic function arcsine transformation for each marker, and lymphocyte gating. In treating cytometric data as a probability distribution, pretreatment can be potentially an artifact. Therefore, we decided to perform only minimal pretreatment in our study design. Our lymphocyte gating process selected CD19-positive or CD138-positive cells in the case of B cell FACS, and CD3-positive cells in the case of T cell FACS, which extracted the lymphocytes. An example of this lymphocyte gating is shown in Supplementary File S1. The preprocessed data are the same as in the preprint paper of our previous work [11].

We used the SNP genotype data from our previous paper [26]. For the SNP genotype data, 1,665,663 SNP genotypes on the autosomes of 298 people were used, satisfying the minor allele frequency (MAF) > 0.01 and the Hardy–Weinberg equilibrium test *P* value of >1.0 × 10^−7^. Although MAF > 0.05 was used in ref. [26], in this study we used MAF > 0.01 so that we included more SNPs in the analysis. The annotation of the SNPs was performed by the web-based tool SNPnexus [27] based on GRCh38 and gene annotation in the Ensembl database.

### Comprehensive quantification of cell subset fractions

First of all, in order to describe how broad lymphocyte subset populations differ over the course of vaccination, we conducted comprehensive quantification of lymphocyte subsets, using an automatic approach with a parametric model (detail method in Supplementary File S2). For each marker of each FACS data, a cutoff value for determining positive/negative was calculated. We excluded IgM and CD138 in the B cell FACS dataset from this analysis, and all remaining markers showed a bimodal distribution. CD3 in the T cell FACS dataset was also excluded because we had selected CD3-positive cells in the lymphocyte gating process. Then, the abundance of 2^4^ = 16 subsets in B cell FACS and 2^6^ = 64 subsets in T cell FACS were quantified, using the positive/negative combination of all cells defined by the cutoff values. The percentage of the total number of cells for each subset was calculated to obtain a cell ratio matrix with the number of samples × the number of subsets. At each time point, changes before and after vaccination were tested using a Wilcoxon signed-rank test, and a subset with FDR *Q* value <0.01 was searched.

### Embedding lymphocyte profiles into Euclidean space

**Fig. 1:**
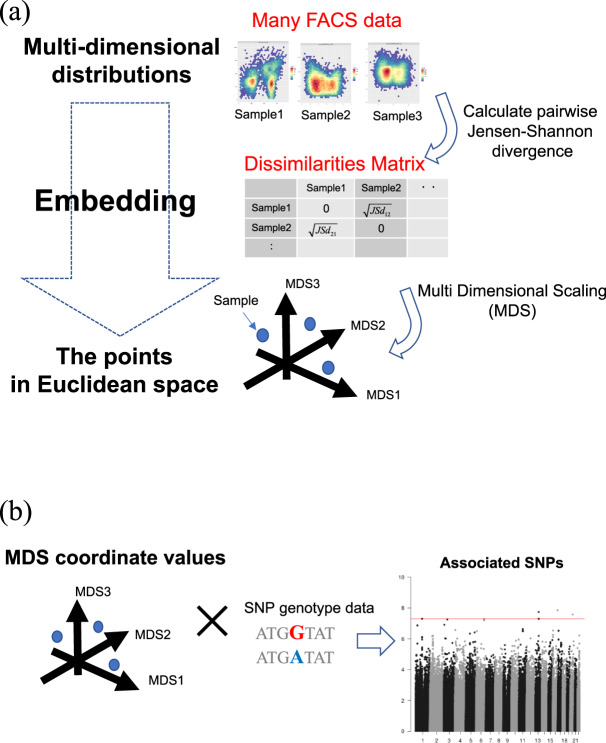
Outline of embedding a FACS dataset into a low-dimensional space. **a** The procedure for extracting feature statistics from FACS data and embedding them with multidimensional markers to Euclidean space is as follows. We estimated the probability distributions of multiple marker expressions of each FACS dataset, the square root of the Jensen–Shannon distance between these distributions was estimated, and a sample × sample dissimilarity matrix was constructed. By applying MDS to this dissimilarity matrix, all data were embedded in the low-dimensional Euclidean space that best reflected the relation between samples in terms of their dissimilarity. **b** We considered an MDS coordinate value as trait and analyzed the association between MDS coordinates and SNP genotype data

In order to conduct a GWAS of the individual differences of human lymphocyte profiles, we obtained the feature statistics using cytometry data in a data-driven and nonparametric manner. The procedure for extracting feature statistics from FACS data and embedding them with multidimensional markers to Euclidean space was as follows (Fig. 1a). First, an equally spaced *m* grid was set for each marker expression value. *m* = 10 and *m* = 8 were used in B cell FACS and T cell FACS, respectively. First, we decided the range of each marker. For each sample, we calculated the 5th percentile and 95th percentile of each marker expression, and used the range of each marker between the minimum 5th percentile value and maximum 95th percentile value among all samples. By dividing these ranges into *m* parts, we decided *m*^*n*^ lattice points where *n* is the number of markers. Discrete approximation of the probability density function of a multidimensional distribution for these lattice points was calculated using the *k*-nearest neighbor method with *k* = 60. Normalization was performed so that the sum of each grid was 1, which is the estimated probability mass function of the FACS data. Next, the square root of the Jensen–Shannon distance [28] between the population distributions was estimated. The square root of the Jensen–Shannon distance is a distance metric between probability distributions. Jensen–Shannon distance is defined by the KL divergence and can be written as follows:

$${\mathrm{JS}}\left( {p||q} \right) = \frac{1}{2}\left( {{\mathrm{KL}}\left( {p||\frac{{p + q}}{2}} \right) + {\mathrm{KL}}\left( {q|\frac{{p + q}}{2}} \right)} \right).$$

As a result, a sample × sample dissimilarity matrix was constructed. By applying MDS to this dissimilarity matrix, all cytometry data were embedded into the low-dimensional Euclidean space that best reflected their dissimilarity. The number of meaningful MDS coordinates was determined based on the elbow of the eigenvalue plot. In this research, the top *K* MDS coordinates were defined as the meaningful coordinates:

$$K = _i^{\arg \,\min }\left( {\left| {{\mathrm{Eig}}_{i + 1} - {\mathrm{Eig}}_i} \right| - \left| {{\mathrm{Eig}}_i - {\mathrm{Eig}}_{i - 1}} \right|} \right) - 1,$$

where *i* takes integer values from two to the number of samples −1, and Eig_*i*_ is the *i*th largest eigenvalue. The selected meaningful MDS coordinates were used for subsequent analysis.

We used these MDS coordinate values as the lymphocyte profile feature statistics. However, the biological significance of these feature statistics is still unclear. Using a cell ratio matrix calculated with the parametric model, we examined which lymphocyte subsets the MDS coordinates explain. For all pairs of MDS coordinates and lymphocyte fractions, we calculated the Kendall’s correlation coefficient and *P* value. We then considered MDS coordinates as traits, and analyzed the association between MDS coordinates and SNP genotype data (Fig. 1b). These analyses identified genetic variations with whole lymphocyte profile differences.

### GWAS for the MDS coordinates

To examine the genetic effects of the lymphocyte profiles, a GWAS was performed on the MDS coordinate values, and SNPs that were significantly related to these feature statistics were examined. Calculations were performed by linear regression for each SNP using the software PLINK v1.07 [29]. The target traits are the coordinate values of MDS1, MDS2, MDS3, MDS4, and MDS5 in B cell FACS, and MDS1 and MDS2 in T cell FACS at each of Days 0, 1, 7, and 90. In the case of Day 0, the following linear regression model was used:

$${\mathrm{MDS}}_i = b_0 + b_1\,{\mathrm{SEX}} + b_2\,{\mathrm{AGE}} + b_3\,{\mathrm{SNP}} + b_4\,{\mathrm{GROUP}} + {\mathrm{error}},$$

where SNP represents the SNP allele count, SEX and AGE are the covariates, and the error term is the random error under a normal distribution. GROUP is also a covariate that takes one of two groups and represents a batch effect. In the case of Days 1, 7, and 90, we added the MDS coordinates value from Day 0 (BASELINE) as a covariate to the model, and the following linear regression model was used:

$${\mathrm{MDS}}_i =	 \, b_0 + b_1\,{\mathrm{SEX}} + b_2\,{\mathrm{AGE}} + b_3\,{\mathrm{SNP}} + b_4\,{\mathrm{GROUP}}\\ 	 + b_{\mathrm{5}}\,{\mathrm{BASELINE}} + {\mathrm{error}}.$$

In the case of Days 1, 7, and 90, the following model with the value at Day 0 added as a baseline covariate was used. *P* values were calculated for each SNP. Using 50,000 randomly picked SNPs, we visualized the correlation of the regression coefficients as beta and *P* values between MDS coordinates with the python matplotlib library.

For each day of B cell FACS and T cell FACS, we integrated the *P* values of MDS coordinates into one representative value using the meta-analysis method. The differences of the lymphocyte profiles were retained by the Euclidean distance on the MDS coordinate system. We considered that the effects of the SNPs are represented as a vector on the MDS coordinate system, which is unlikely to be orthogonal to a particular MDS coordinate. Then, we integrated the *P* values based on the maximum *P* values, assuming that the candidate SNPs for differences in lymphocyte profiles were associated with all MDS coordinates at 1-day point. We used the maximump function in the R package “metap” for this procedure [30]. Also, we calculated the genomic inflation factor (*λ*) based on median chi-squared values. When *λ* is almost equal to 1 (for example, *λ* < 1.1), the population structure is considered to be subtle [31]. The results of the GWAS were visualized by a Manhattan plot, which was drawn by the R package “qqman” [32]. We identified the SNPs passing the global significance line (*P* < 5.0 × 10^−8^) and the stringent significance threshold (*P* < 6.25 × 10^−9^), which is global significance divided by 8 because we used eight traits (2 dataset × 4 time points) considering multiple testing burden. We considered the SNPs passing the stringent significance threshold as the significant SNPs and the SNPs which wasn’t passing the stringent significance threshold, but passing global significance line as the suggestive SNPs. We downloaded the previously reported SNPs with “response to vaccine” from the GWAS catalog database on September 7, 2020 and compared our results with them. Next, we obtained the gene annotations of our SNPs with the SNPnexas tool [27] to examine the function of the annotated genes in the significant and suggestive SNPs. To select biologically important genes and their networks from the SNP-annotated gene set, we used the STRING database version 11.0 to depict annotated gene networks [33], and extracted the genes with at least one link and their protein–protein interactions.

## Results

### Comprehensive quantification of cell subset fractions

Figure S1 shows the comprehensive change of the B cell subsets and T cell subsets of Days 1, 7, and 90 from Day 0. The CD19−IgD−CD21−CD27+ subset was relatively increased after vaccination. That subset is considered to contain plasma cells. The CD19+IgD+CD21+CD27− subset was relatively decreased at the Day 7 profile and recovered at Day 90. That subset can be annotated as the naive B cell subset. In the case of T cell FACS, the number of CD45RO-negative cells decreased, and the number of CD45RO-positive cells increased, which corresponds to the fact that the naive population differentiates into an effector/memory population. These results correspond to immunological knowledge. However, with this parametric model, it is difficult to comprehensively describe all the changes occurring after vaccination. Supplementary Files S3 and S4 show density plots of B cell FACS data and T cell FACS data for Person ID 1, and the cutoff value for each marker as an example of representative data. Also, the median value of each lymphocyte subset among 301 individuals at each time point is shown in Figs. S2 and S3. The box whisker diagram of all B cell subsets and T cell subsets is shown in Supplementary Files S5 and S6. Note that the quantification results of rare subsets are subject to automatic preprocessing, and quantification artifacts, such as dead cells are not removed.

### Embedding the lymphocyte profiles to a Euclidean space by MDS

**Fig. 2:**
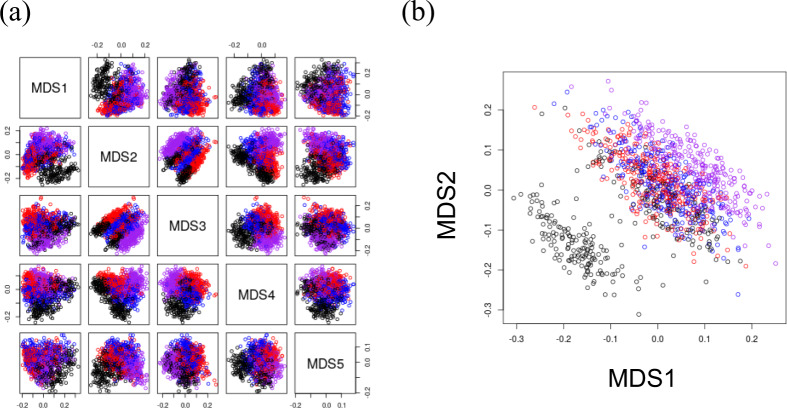
The paired MDS coordinate plots for MDS1, MDS2, MDS3, MDS4, and MDS5 in the case of the B cell FACS dataset (**a**), and MDS1 and MDS2 in the case of the T cell FACS dataset (**b**). The points colored red, blue, black, and purple represent Days 0, 1, 7, and 90 samples, respectively

All cytometry data from B cell FACS and T cell FACS were embedded in a low-dimensional Euclidean space that best reflected their dissimilarity. We call the MDS coordinates with the *i*th largest eigenvalue in B cell FACS dataset and T cell FACS dataset as B_MDSi and T_MDSi, respectively. Figure S4 shows a plot of the top eigenvalues in B cell FACS and T cell FACS. From the eigenvalue plot, up to MDS5 of B cell FACS and up to MDS2 of T cell FACS were adopted as significant eigenvalues. They were used for the subsequent analysis as meaningful coordinates. Figure 2 shows a co-plot of the coordinates of 1173 samples in B cell FACS and T cell FACS. In the case of both B cell FACS and T cell FACS, the samples at different time points are separated on the MDS coordinates. Time-series information is consistently separated as in past studies [6–8]. It was confirmed that the MDS coordinate value is appropriate as a representative variable of the lymphocyte profile. And because the batch effect of cytometry data (Groups A and B) affects the MDS coordinate values (Fig. S5), we decided to consider this batch effect in the following GWAS analysis. In addition, it was suggested that multiple MDS coordinates not only explain time-series information, but also include other genetic or environmental factors that cause individual differences in the coordinate values.

Table S1 has the correlation coefficient and *P* value of all paired MDS coordinates and lymphocyte subset fractions quantified with the parametric models, and Table S2 shows the 28 pairs with |correlation coefficient| > 0.25 and Bonferroni-corrected *P* < 0.05 that were extracted. The explanation of each MDS coordinate is written in Supplementary File S7. This result provides clues associating a subset of lymphocytes that interpret the meaning of each MDS coordinate.

### GWAS for MDS coordinates

#### Genetic features of the individual differences in human lymphocyte profiles

To examine the genetic effects of the lymphocyte profile, a GWAS was performed on MDS coordinate values, and SNPs that were significantly related to these feature statistics were examined. The target traits are the coordinate values of MDS1, MDS2, MDS3, MDS4, and MDS5 in B cell FACS, and MDS1 and MDS2 in T cell FACS at Days 0, 1, 7, and 90, respectively. While the GWAS for Day 0 identified SNPs associated with steady-state lymphocyte profiles, the GWASs for Days 1, 7, and 90 identified SNPs associated with individual differences in immune responses at each time point after vaccination. We searched the literature for these SNPs. The QQ plots for Days 0, 1, 7, and 90 on each MDS coordinate are shown in Fig. S6.

Population structuring was considered to have little effect on GWAS results, because the genomic inflation factors were almost all 1 in all analyses (ranged from 1 to 1.01426).

**Fig. 3:**
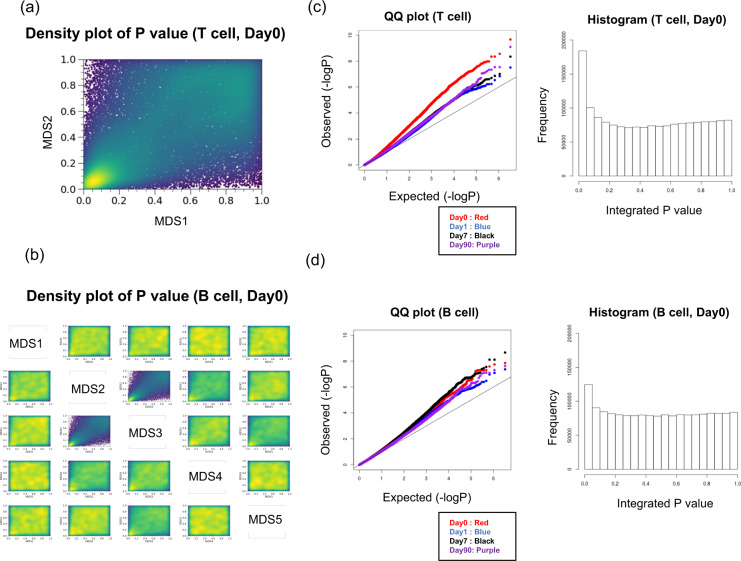
Genetic features of T cell and B cell profiles derived from the GWAS. **a** Density plot of the *P* values of T_MDS1 and T_MDS2 2 (Day0). **b** Density plot of the *P* values of B_MDS1, B_MDS2, B_MDS3, B_MDS4, and B_MDS5 2 (Day0). **c** QQ plot and histogram of the integrated *P* values in the T cell FACS. **d** QQ plot and histogram of the integrated *P* values in the B cell FACS

Figure 3a shows a density plot of *P* values for T cell MDS coordinates at Day 0. SNPs were observed at high densities in both MDS coordinates and regions with low *P* values. In the same plot showing the result of T cell FACS data at other day points and B cell FACS data, a similar trend was observed for most MDS coordinate pairs (Fig. 3b and Figs. S7–S10). Figure S11 also shows a density plot of the values of the regression coefficients of all the MDS coordinates (B_MDS1, B_MDS2, B_MDS3, B_MDS4, B_MDS5, T_MDS1, and T_MDS2) on Day 0. Interestingly, the pair of B_MDS2 and B_MDS3 showed the largest correlation coefficient of the regression at 0.82, while the correlation coefficient of these coordinate values was 0.16. In the other day points, the beta values of B_MDS2 and B_MDS3 showed a high correlation (Figs. S12–S14). From these results, we found that the SNPs associated with lymphocyte profiles were associated with multiple MDS coordinates. These results are valid because the differences in lymphocyte profiles were quantified as Euclidean distances on the MDS coordinate system, and some MDS coordinate values at the specific day points were correlated. The beta values of B_MDS2 and B_MDS3 showed a very high correlation coefficient overall, although there was also some correlation between those coordinate values. This suggests that B_MDS2 and B_MDS3 may be affected by common genetic effects.

After the integration based on the maximum *P* value, we generated histograms and QQ plots with integrated *P* values of T cell FACS and B cell FACS, as shown in Fig. 3c, d, respectively. The histograms show that the distribution of post-integration *P* values using the maximum *P* value takes the form of a mixture distribution of the uniform distribution from the SNP sets, which is irrelevant to the lymphocyte profile and the other distributions from the SNP sets that are associated with MDS coordinate space in all cases. The QQ plots show that the *P* value after integration deviated from the uniform distribution in all cases. These deviations suggested that a large number of SNPs had a small effect on the differences in the lymphocyte profiles. It is considered that this is a general feature in the genetics of vaccination response, since QQ plots with similar characteristics were obtained in a past GWAS that used cytokine amounts as a trait [3]. This feature is common to the B cell profile and the T cell profile. However, the QQ plot on the T cell on Day 0 was especially deviated from the uniform distribution, and these weren’t shown in the QQ plot of the B cell profile. The T cell profile in the steady state is affected more strongly by the SNPs, but the genetic effect on the T cell profile after vaccine intervention may be reduced.

#### Candidate SNPs or genes explaining the individual differences in human lymphocyte profiles

SNP level functional annotation in our GWAS identified seven significant SNPs (*P* < 6.25 × 10^−9^) and the 36 suggestive SNPs (6.25 × 10^−9^ < *P* < 5.0 × 10^−8^) associated with either trait. The Manhattan plots are shown in Figs. S15 and S16. Table 1 shows the total 43 significant and suggestive SNPs, which we focused on for further analysis. We also searched for the SNPs that have been reported with the trait “response to vaccine” in the GWAS catalog database [34]. A total of 190 SNPs identified in 24 previous studies are registered (the output of the GWAS catalog is shown in Table S3). Although none of the SNPs identified in this research were included in these 190 SNPs, one annotated gene is common *(LPP*). This gene has been reported to be a candidate gene in a GWAS study of cytokine responses to a smallpox vaccine [3].

**Table 1:** SNPs that passed the global significance line in B or T cell FACS

rs ID	Chromosome	Position	REF allele	ALT allele	*P* values	Trait
rs10460510	2	75,697,424	G	A	2.10E−10*	T_day0
rs39426	7	25,106,296	G	A	7.97E−10*	T_day90
rs75504175	3	118,997,705	G	A	2.18E−09*	B_day7
rs3794852	18	74,709,289	C	T	2.68E−09*	T_day0
rs59422776	17	766,399	C	T	4.42E−09*	T_day0
rs118160548	17	771,456	A	G	4.42E−09*	T_day0
rs9919764	12	13,492,704	C	A	4.47E−09*	T_day7
rs12611599	2	208,961,028	C	T	7.73E−09	B_day7
rs12614989	2	208,961,158	T	C	7.73E−09	B_day7
rs60057223	10	8,536,674	A	C	1.05E−08	T_day0
rs11592991	10	8,539,331	G	A	1.05E−08	T_day0
rs10905390	10	8,537,695	A	G	1.20E−08	T_day0
rs10905391	10	8,540,328	G	A	1.20E−08	T_day0
rs2696860	16	86,327,721	A	G	1.41E−08	B_day0
rs6568431	6	106,588,806	A	C	1.45E−08	T_day0
rs6797423	3	126,215,130	G	A	1.55E−08	T_day0
rs9381968	6	13,353,734	T	C	1.64E−08	T_day0
rs9530814	13	79,285,728	T	C	1.82E−08	B_day0
rs169140	16	24,245,090	A	G	2.00E−08	T_day0
rs6736713	2	230,366,542	C	T	2.22E−08	T_day0
rs138332350	1	241,794,224	T	C	2.39E−08	T_day0
rs78509568	2	197,398,200	T	G	2.43E−08	B_day90
rs4587178	6	98,421,991	T	C	2.47E−08	T_day0
rs78760834	17	740,734	G	C	2.50E−08	T_day0
rs58014646	20	41,344,189	A	C	2.61E−08	B_day0
rs4668882	2	15,334,251	G	A	2.83E−08	B_day7
rs55914228	10	126,896,675	A	G	2.87E−08	T_day90
rs7069729	10	126,896,989	A	G	2.87E−08	T_day90
rs6801602	3	188,519,594	A	G	3.00E−08	T_day0
rs8073989	17	15,194,724	T	C	3.08E−08	T_day1
rs2051344	18	74,715,653	G	T	3.13E−08	T_day0
rs4685806	3	4,772,692	T	C	3.47E−08	T_day0
rs35806	10	79,165,830	G	A	3.48E−08	B_day7
rs10436922	1	91,317,700	G	A	3.65E−08	T_day0
rs1040893	6	106,596,087	T	C	4.07E−08	T_day0
rs76280036	5	142,276,784	G	A	4.10E−08	B_day7
rs13344319	19	54,921,227	G	A	4.17E−08	T_day0
rs76425237	12	130,844,567	T	C	4.35E−08	B_day1
rs11920819	3	80,269,036	G	A	4.61E−08	T_day90
rs7427090	3	80,274,761	C	T	4.61E−08	T_day90
rs4932564	15	92,176,277	A	G	4.81E−08	T_day0
rs7639948	3	188,546,497	T	C	4.95E−08	T_day0
rs9851822	3	150,364,364	G	A	4.98E−08	B_day90

These were SNPs associated with individual differences of lymphocyte profiles (Day 0) and their change after vaccination (other than Day 0). Each column represents the following; rs ID of SNP, chromosome, position, reference allele, alternative allele, *P* value of GWAS, B cell FACS/T cell FACS, and Day. *P* values with asterisk (*) indicates it passed the stringent significance threshold (<6.25 × 10^−9^)

In addition, rs6568431 has been reported to be associated with systemic lupus erythematosus (SLE) in multiple studies [35–37], and it has an A > C allele and is situated in the intronic region of *ATG5*. *ATG5* is a gene that plays a major role in autophagy and is also strongly associated with SLE [38, 39]. The GWAS beta values of the A allele of rs6568431 for GWAS T_MDS1_day0 and T_MDS2_day0 of (ATG5) are 0.02 and −0.02, respectively. This suggests that this SNP causes T_MDS1 drifts in the positive direction and T_MDS2 drifts in the negative direction on the T cell MDS space. Table S2 shows that T_MDS1 has a positive correlation with the fraction of CD4+CD8−CD45RA+CD45RO−CD25-CCR7+ (annotated to CD4+ naive T cells), and T_MDS2 has a negative correlation with this subset fraction. In fact, the genotype of this SNP (AA, AC, or CC) is related to the abundance of this subset where the Jonckheere–Terpstra test *P* = 0.0024 using the R package’s clinfun’s jonckheere.test function with the number of permutations set to 10,000 [40]. A box whisker diagram of each genotype is shown in Fig. S17. This SNP may be associated with SLE through individual differences in T lymphocyte profiles, in particular the CD4+ naive T cell subset.

In addition, we searched for our SNPs in our previous eQTL study using the same genotype data [26], the blood eQTL browser [41], and the eQTL database of the GTEx Consortium [42]. While none of the SNPs were common between our previous eQTL study and this study, a total of 14 out of 43 SNPs were found in the other two previous studies (Table S4). Given that many organs are involved in the immune response, the SNPs identified in this study may influence individual differences in lymphocyte profiles through the expression of these genes.

**Fig. 4:**
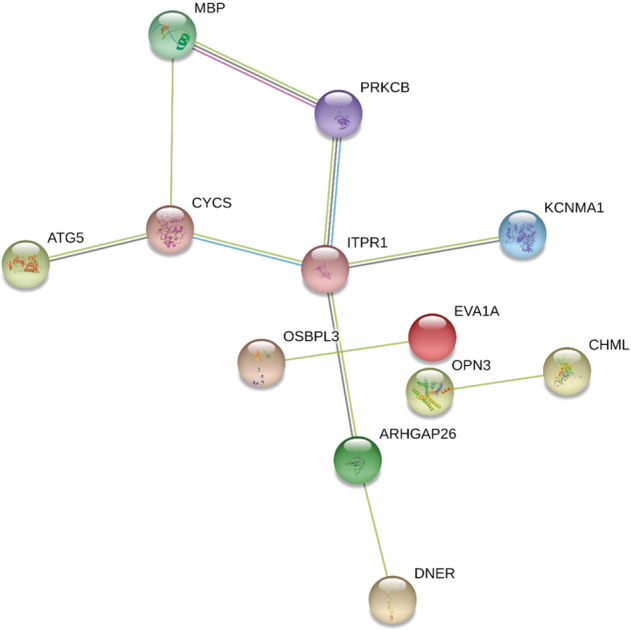
Protein–protein networks of GWAS genes with at least one interaction in the STRING database. The color of the edge represents the type of interaction as defined in the STRING database. (Black: coexpression; purple: experimentally determined interaction; light blue: database annotated; and yellow: automated text mining). Genome-wide association study of individual differences of human lymphocyte profiles using large-scale cytometry data

Next, we obtained the gene annotations of our SNPs with the SNPnexas tool to examine the function of the annotated genes in the GWAS. Table S5 shows the gene annotations of all 43 SNPs as the output of SNPnexas. To select biologically important genes and their biological links, we used the STRING database to depict annotated gene networks. Figure 4 shows the network of 12 genes with at least one link (*ITPR1*, *OPN3*, *DNER*, *CYCS*, *ATG5*, *OSBPL3*, *MBP*, *PRKCB*, *CHML*, *ARHGAP26*, *KCNMA1*, and *EVA1A*). The gene that is connected to the largest number of genes in the network is *ITPR1*. rs4685806 is situated in the intronic region of this gene. The *CYCS* gene is annotated to rs39426 with the second lowest GWAS *P* value, which is situated 53 kbp downstream of the end of this gene. This gene has been reported to be associated with SLE in a previous GWAS [37]. The *PRKCB* gene has a role in both oxidative stress induced autophagy and B cell activation [43, 44]. rs169140 is situated 13 kbp upstream of the end of this gene. The *EVA1A* gene, annotated to rs10460510 with the lowest GWAS *P* value, is situated in the intronic region of this gene. This gene is reported to mediate both autophagy and apoptosis [45, 46]. A series of results suggests that mechanisms related to autophagy and SLE are associated with individual differences in lymphocyte profiles and their change after vaccination.

## Discussion

When the phenotype takes the form of a point cloud from a distribution, such as a FACS result, it is difficult to analyze it with other variables. To analyze these distributions with regular variables with conventional statistical methods, the distribution should be expressed as a vector. In this study, using inter-distribution divergence and MDS, we extracted the independent feature statistics that best explained the differences in the overall lymphocyte profile. Lymphocyte profiles change dynamically with vaccination, and are difficult to describe completely. In addition, a small subset of lymphocytes has been suggested to play an important role in the immune system. It is also difficult to fit a parametric model, such as a bimodal mixture normal distribution model, with some markers. The MDS coordinate values are however effective data-driven and non-parametric feature statistics of the whole lymphocyte profiles.

The candidate genes we report in this study include those that have been reported in the literature to be related to immune phenotypes. We thus considered that we can identify novel candidate genes for individual lymphocyte profile differences and their changes after vaccination that previous GWASs, using conventional immune response biomarkers, such as titers and cytokines could not detect. Recently, personalized medicine, which takes into account such individual differences, has attracted increasing attention in regard to viral immune response or vaccine safety [47]. Unfortunately, the results of this study do not directly predict vaccination response or effectiveness, and are not sufficient to apply to personalized medicine. A detailed future study of the SNPs or annotated genes identified in this study may help to elucidate the molecular basis of individual differences in the immune response, as well as help to develop genomic markers to predict vaccination responses in individuals. In addition, interest in individual differences in response to viral infection has increased due to the COVID19 pandemic. The genetics of individual differences in response to vaccination as identified here could be a meaningful basis for further study.

In this study, using data-driven extraction of lymphocyte profile feature statistics, we combined cytometry data with SNP genotype data by positioning the cytometry data as one layer of a multi-omics approach. Multi-omics analysis combining multiple omics resources has become a highly useful approach and has successfully revealed various biological phenomena [48]. Our approach has enabled us to integrate cytometry data into a multi-omics analysis, which can contribute to the understanding of a complex biological system.

This study has some limitations when interpreting the results biologically. First, this study used relatively few samples. While this study and past GWAS QQ plots suggest the small involvement of many genes in the vaccine response, only 43 SNPs could be detected in our GWAS. We consider that the small sample size caused relatively large *P* values considering the load of multiple testing due to conducting GWAS for eight traits (B/T cell profiles by four time points). This is likely the reason why only one gene (*LPP*) was common between our GWAS and the previously reported genes in the GWAS catalog. Our GWAS probably missed many other candidate SNPs and genes reported in past GWAS. Second, it is unclear what kinds of functional SNPs the workflow used in this study captured. A relatively small number of SNPs were common between those identified in this study and those in previously reported eQTL studies. The method of this study may tend to detect SNPs associated with differences in the overall distribution of protein expression levels that are the combinatorial phenomenon of multiple proteins in pathways. This means that the eQTL analysis did not seem to be able to capture the heterogeneity, because eQTL is a transcriptome analysis of individual genes using bulk cells as samples, so heterogeneity cannot be captured by eQTL analysis using bulk transcriptome data.

The following four points can be listed as improvements in the workflow used in this study. In this study, the MDS coordinate with the smallest eigenvalue was excluded from the analysis. Although this does not explain much of the difference in cell population profiles, such a coordinate is not necessarily immunologically meaningless. Second, it is difficult to consider the batch effect of cytometry data because those data are a point cloud distribution. In this regard, the workflow used in this study can be improved. Third, when the number of samples of cytometric data is limited, the detection power of GWAS becomes small. The development of methods to improve statistical power will be a major improvement. Finally, the workflow used in this study can be applied not only to cytometry data, but also to single-cell RNA-seq (scRNA-seq) data. In recent years, scRNA-seq has been used not only for immunology, but also in various other biological fields [49]. Since scRNA-seq data have higher dimensions than cytometry data, it will be necessary to develop steps for selecting markers for application. Modifying this workflow for scRNA-seq is an issue we will address in the future.

In this study, we estimated the distribution of lymphocyte profiles in peripheral blood from FACS data and extracted feature statistics. With the GWAS, we were able to identify SNPs related to differences in lymphocyte profiles. The workflow of this study is considered to be a powerful approach to data-driven identification of biological factors involved in the complex biological phenomena and diseases based on cell population profiles.

## Supplementary information

Table S1

Table S2

Table S3

Figure S1

Figure S2

Figure S5

Figure S7

Figure S8

Figure S9

Figure S10

Figure S11

Figure S12

Figure S13

Figure S14

Figure S15

Figure S16

Figure S17

File S1

File S2

File S3

File S4

File S5

File S7

File S6

Figure S3

Figure S4

Figure S6

Table S4

Table S5

## Data Availability

FACS data and SNP genotype data are available under the condition of collaboration, because they are the resources of on-going studies. Please contact us for details on their availability.
