# Supplementary figures and images for: Genome-wide association study of individual differences of human lymphocyte profiles using large-scale cytometry data
Source: J Hum Genet. 2020 Nov 23;66(6):557–67. doi: 10.1038/s10038-020-00874-x (PMC8144016; doi:10.1038/s10038-020-00874-x)

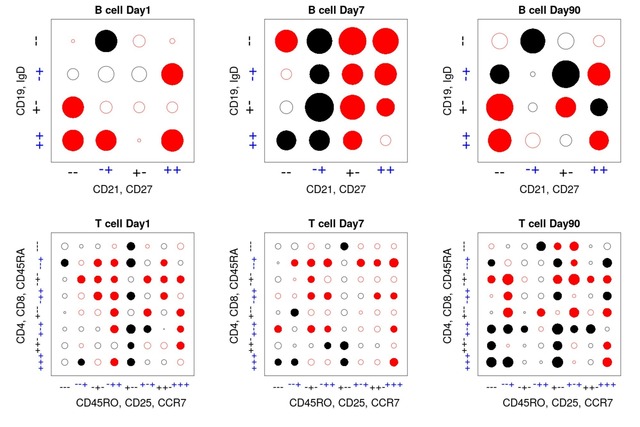

Supplement: Supplementary file 4 — Figure S1 [file 10038_2020_874_MOESM4_ESM.jpg]

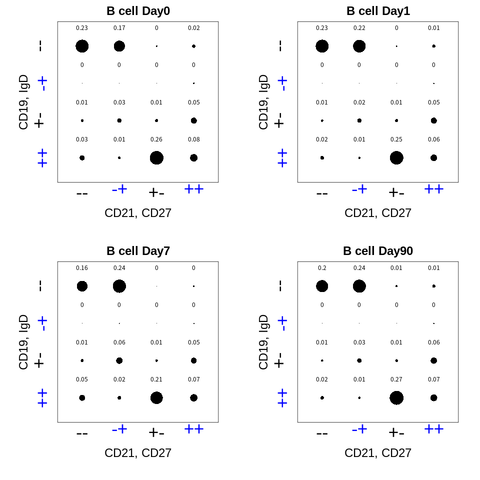

Supplement: Supplementary file 5 — Figure S2 [file 10038_2020_874_MOESM5_ESM.png]

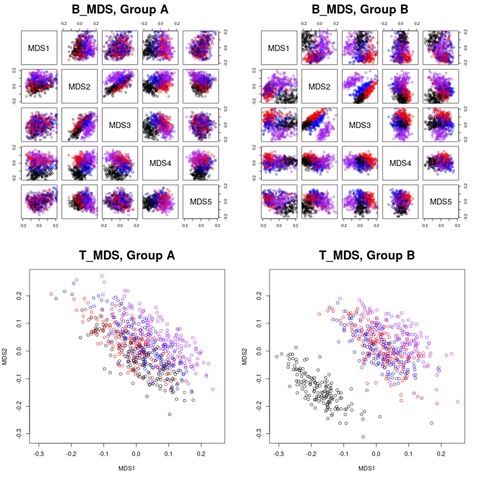

Supplement: Supplementary file 6 — Figure S5 [file 10038_2020_874_MOESM6_ESM.jpg]

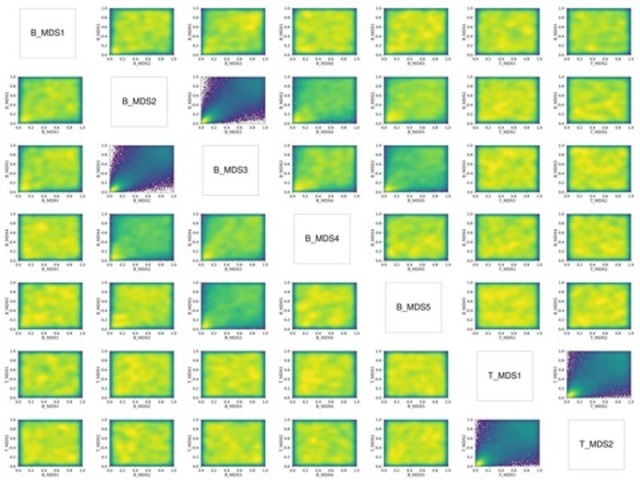

Supplement: Supplementary file 7 — Figure S7 [file 10038_2020_874_MOESM7_ESM.jpg]

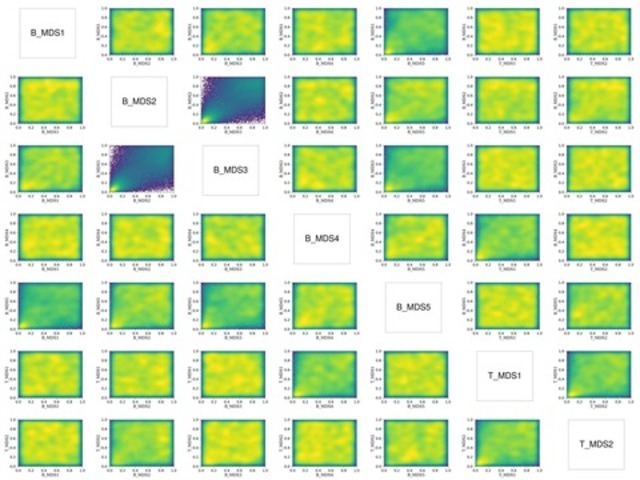

Supplement: Supplementary file 8 — Figure S8 [file 10038_2020_874_MOESM8_ESM.jpg]

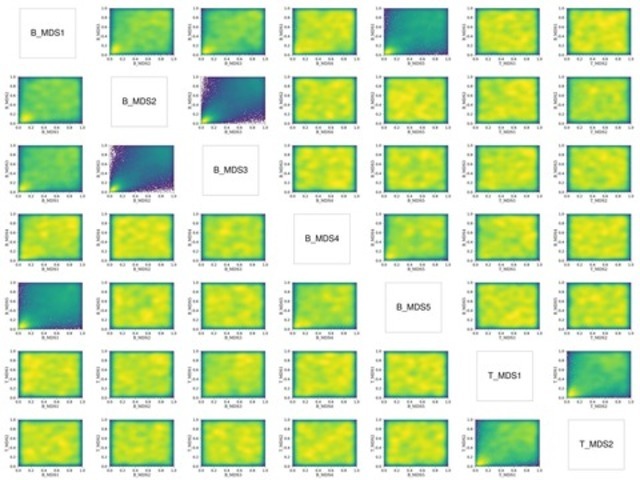

Supplement: Supplementary file 9 — Figure S9 [file 10038_2020_874_MOESM9_ESM.jpg]

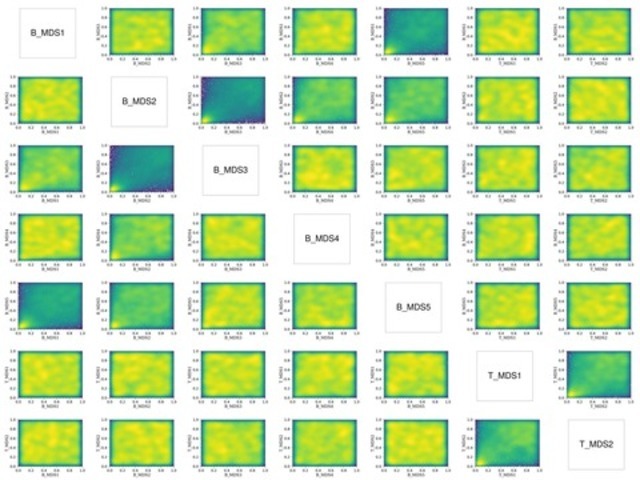

Supplement: Supplementary file 10 — Figure S10 [file 10038_2020_874_MOESM10_ESM.jpg]

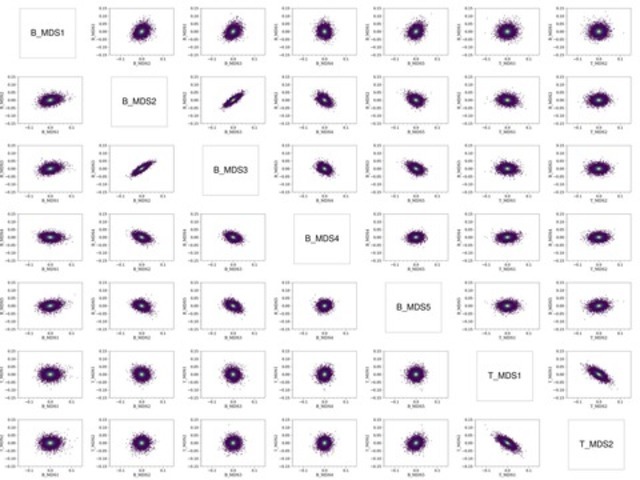

Supplement: Supplementary file 11 — Figure S11 [file 10038_2020_874_MOESM11_ESM.jpg]

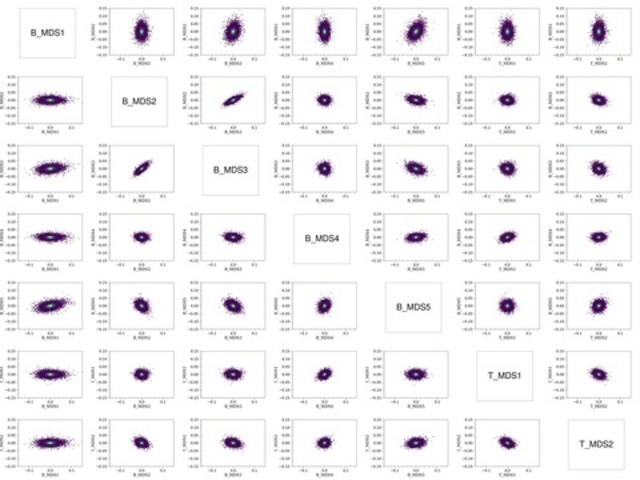

Supplement: Supplementary file 12 — Figure S12 [file 10038_2020_874_MOESM12_ESM.jpg]

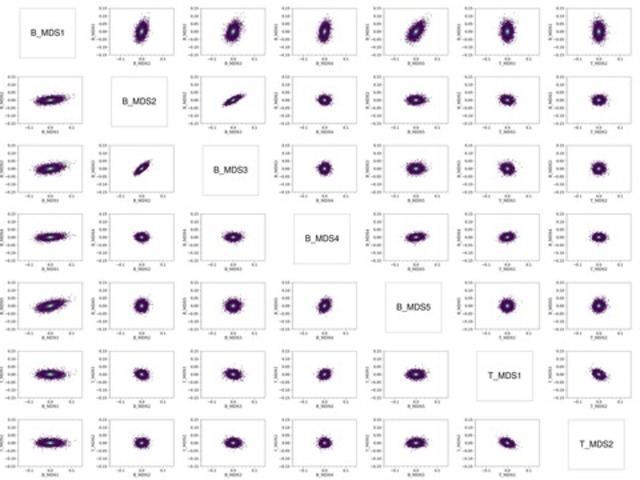

Supplement: Supplementary file 13 — Figure S13 [file 10038_2020_874_MOESM13_ESM.jpg]

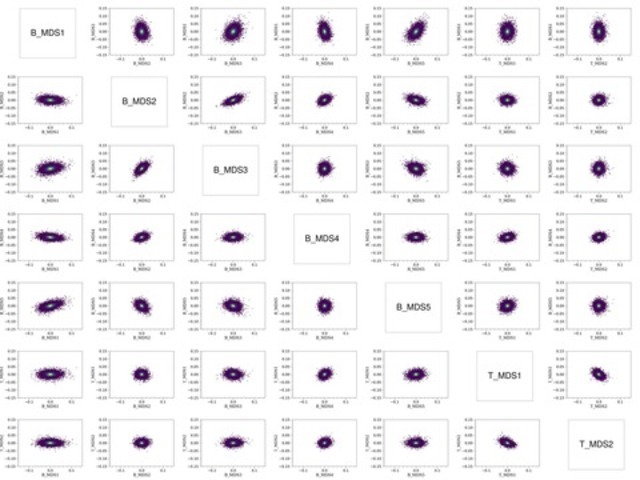

Supplement: Supplementary file 14 — Figure S14 [file 10038_2020_874_MOESM14_ESM.jpg]

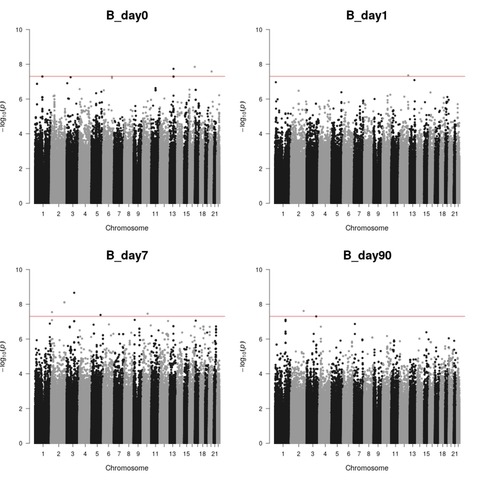

Supplement: Supplementary file 15 — Figure S15 [file 10038_2020_874_MOESM15_ESM.jpg]

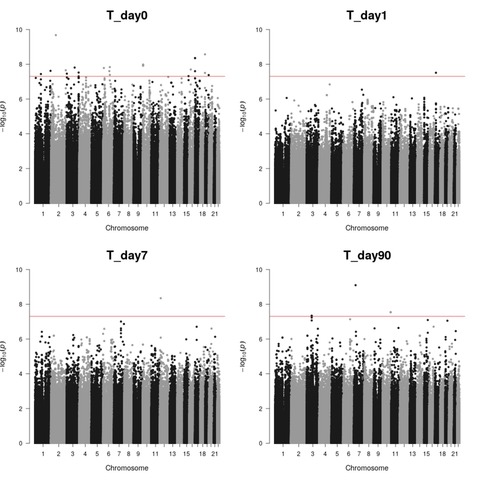

Supplement: Supplementary file 16 — Figure S16 [file 10038_2020_874_MOESM16_ESM.jpg]

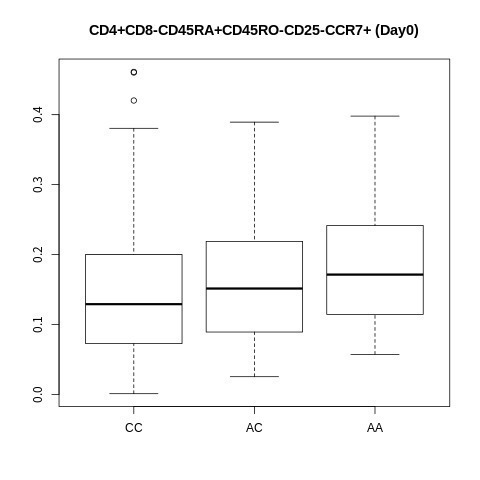

Supplement: Supplementary file 17 — Figure S17 [file 10038_2020_874_MOESM17_ESM.jpg]

B cell FACS Day0

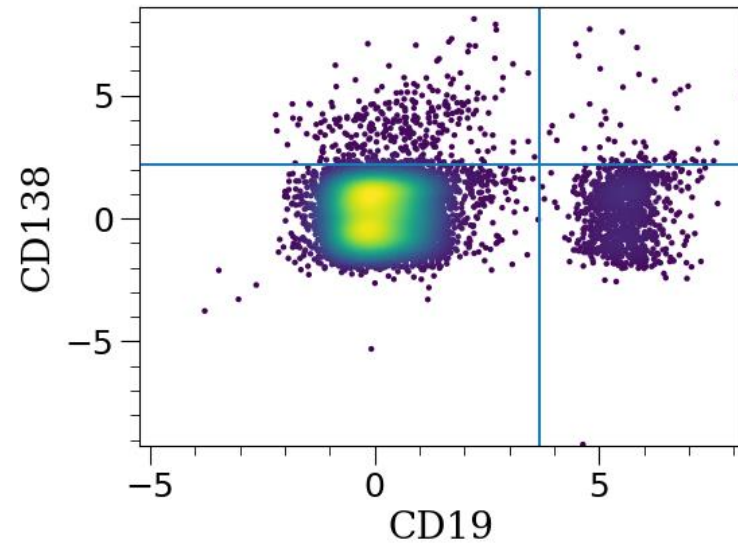

B cell FACS Day1

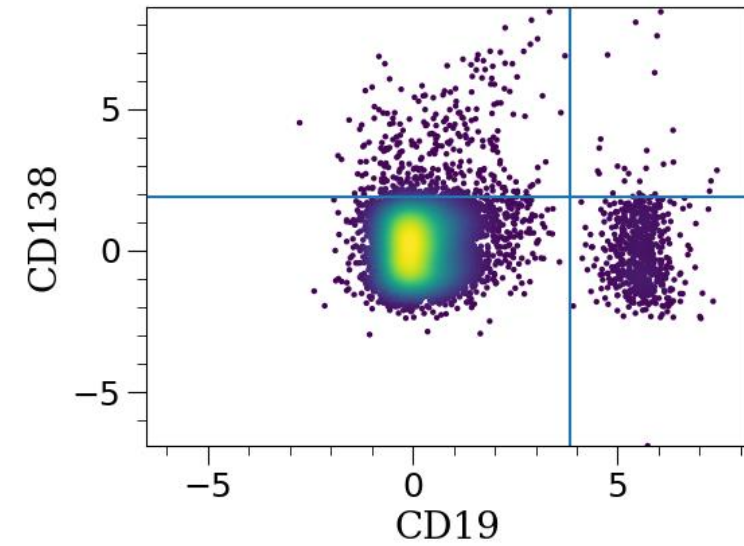

B cell FACS Day7

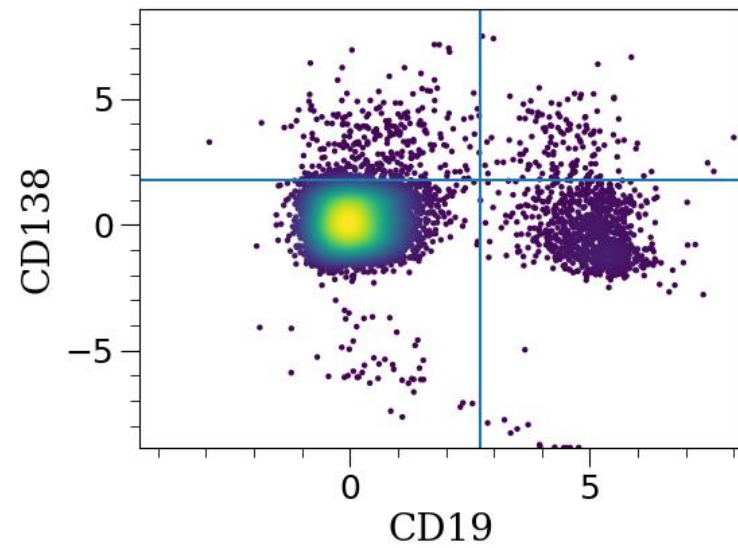

B cell FACS Day90

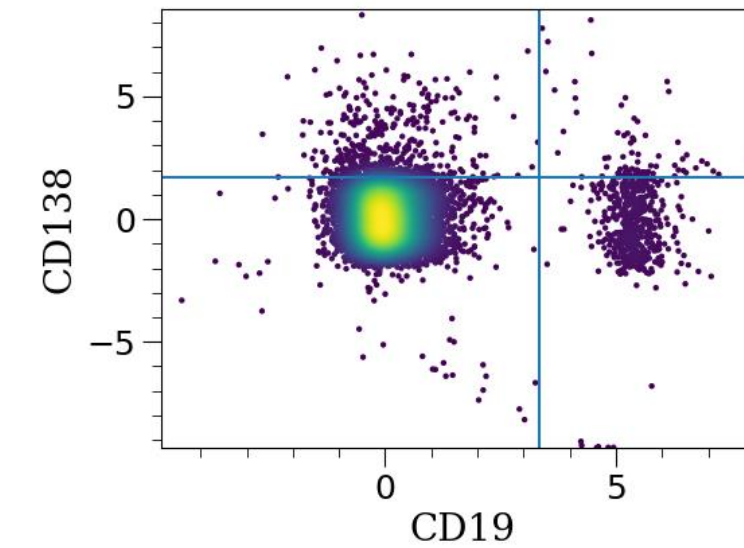

# T cell FACS

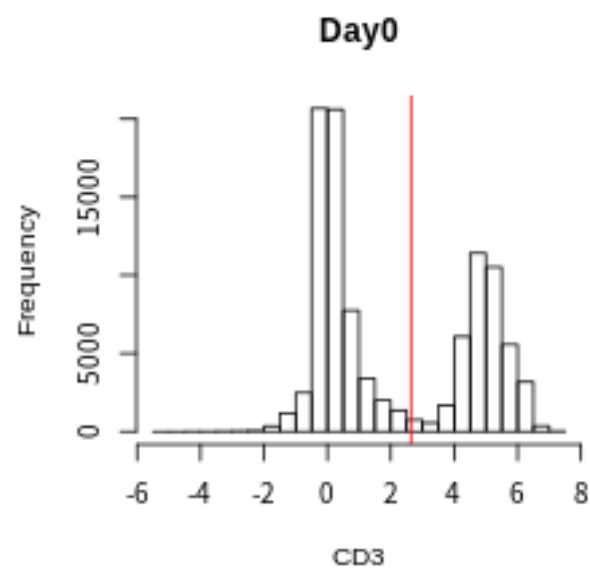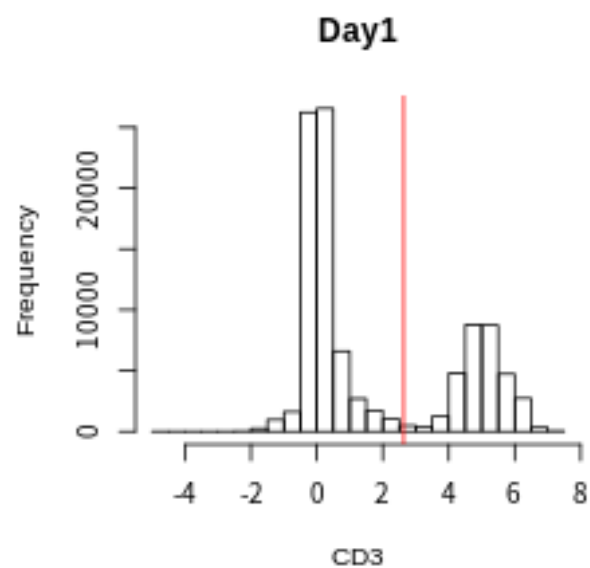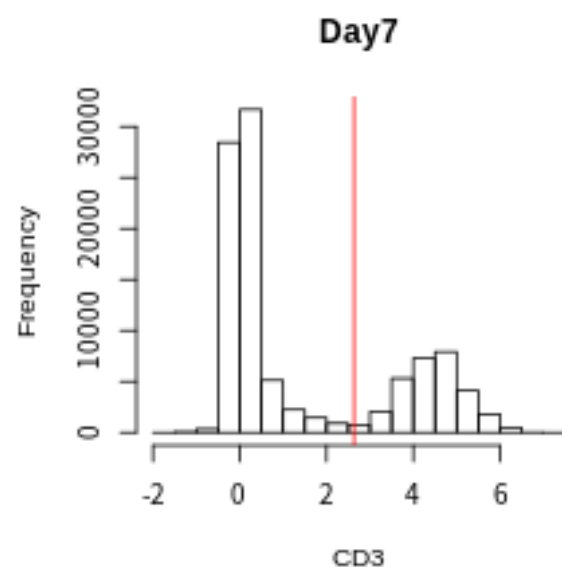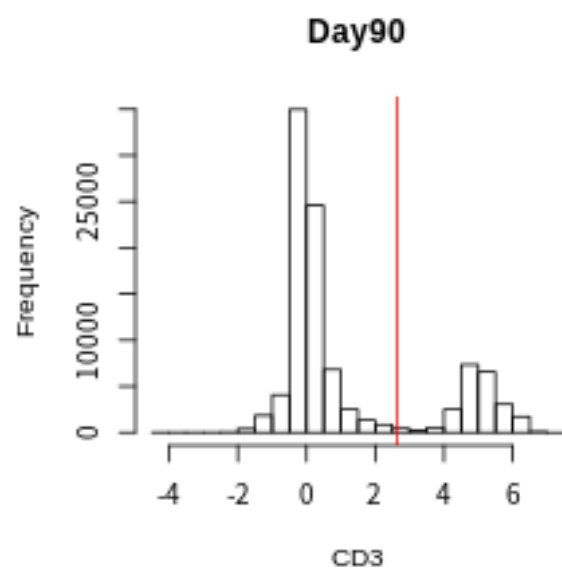

Supplement: Supplementary file 18 — File S1 [file 10038_2020_874_MOESM18_ESM.pdf]

CD19

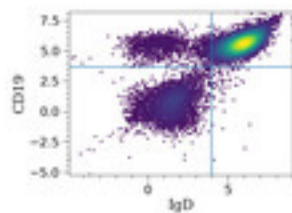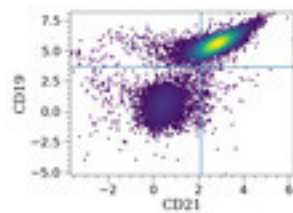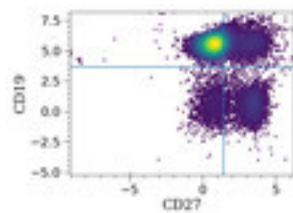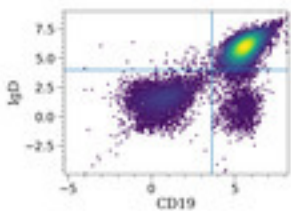

IgD

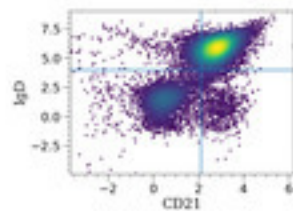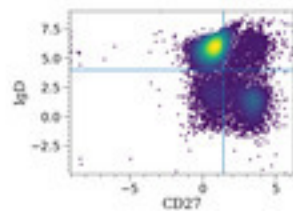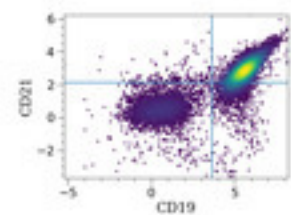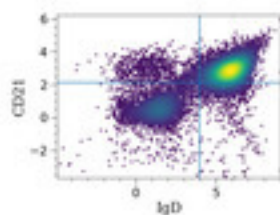

CD21

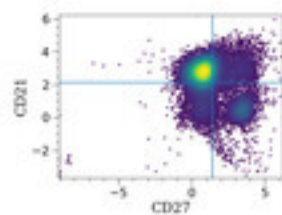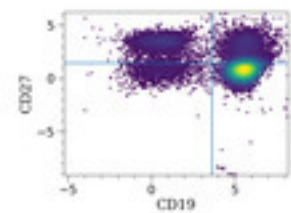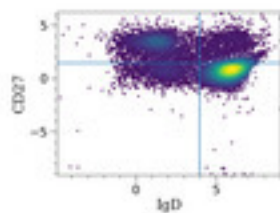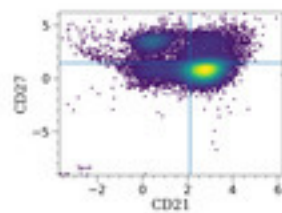

CD27

CD19

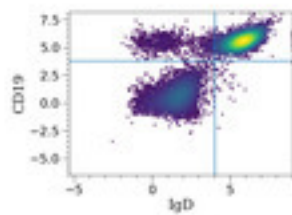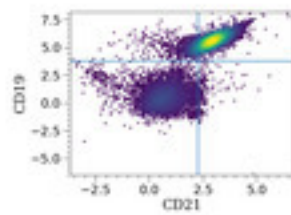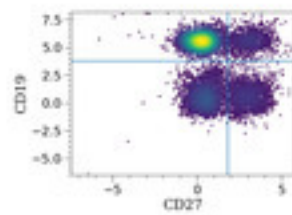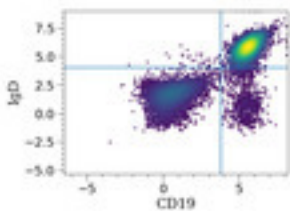

IgD

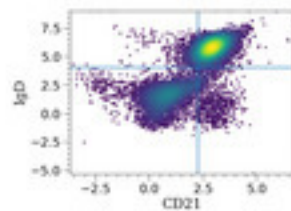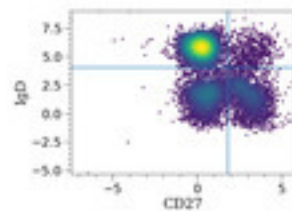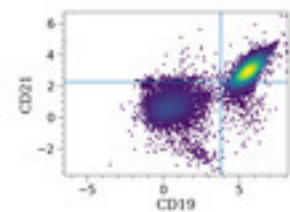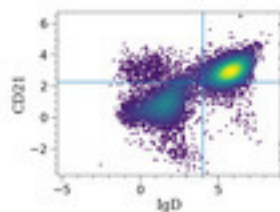

CD21

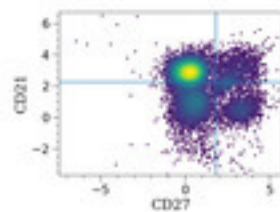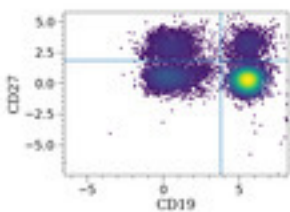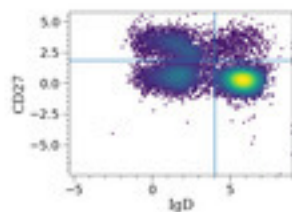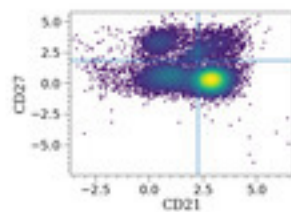

CD27

CD19

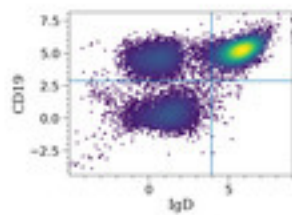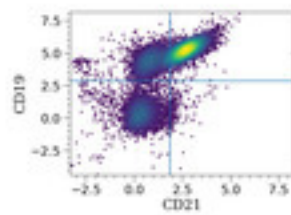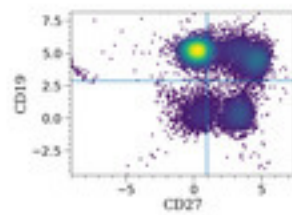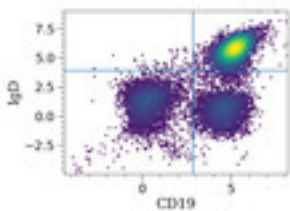

IgD

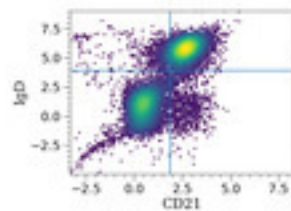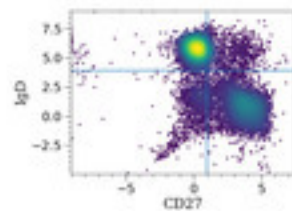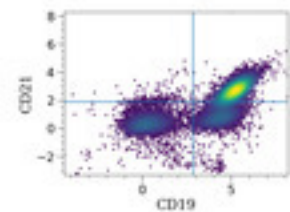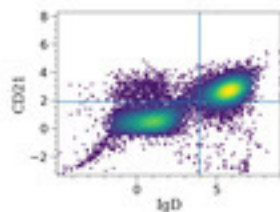

CD21

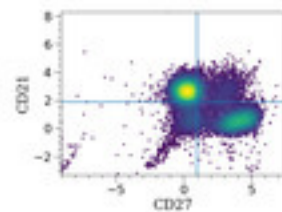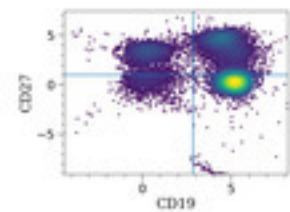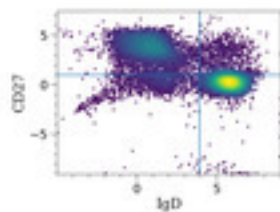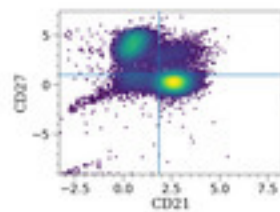

CD27

CD19

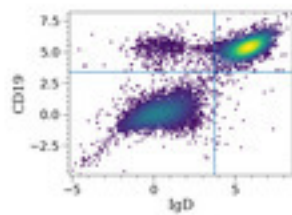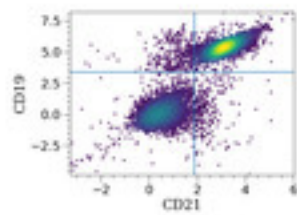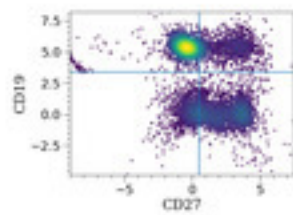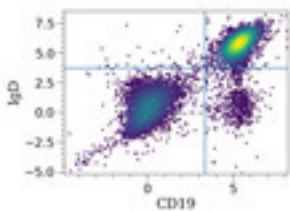

IgD

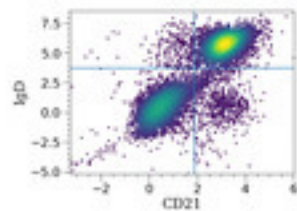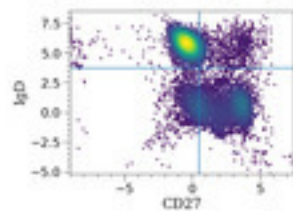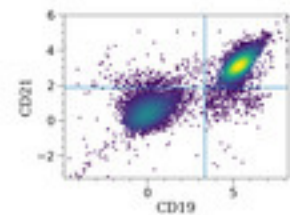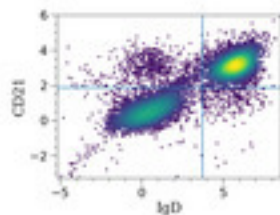

CD21

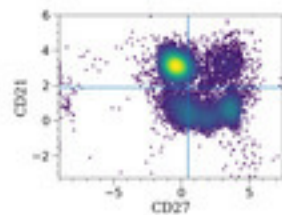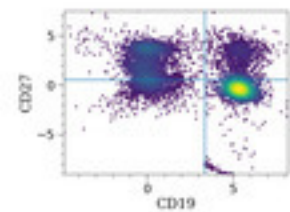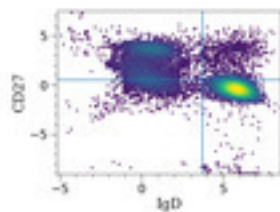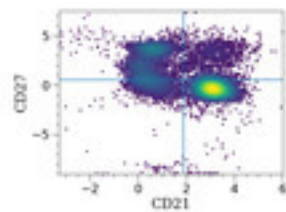

CD27

Supplement: Supplementary file 20 — File S3 [file 10038_2020_874_MOESM20_ESM.pdf]

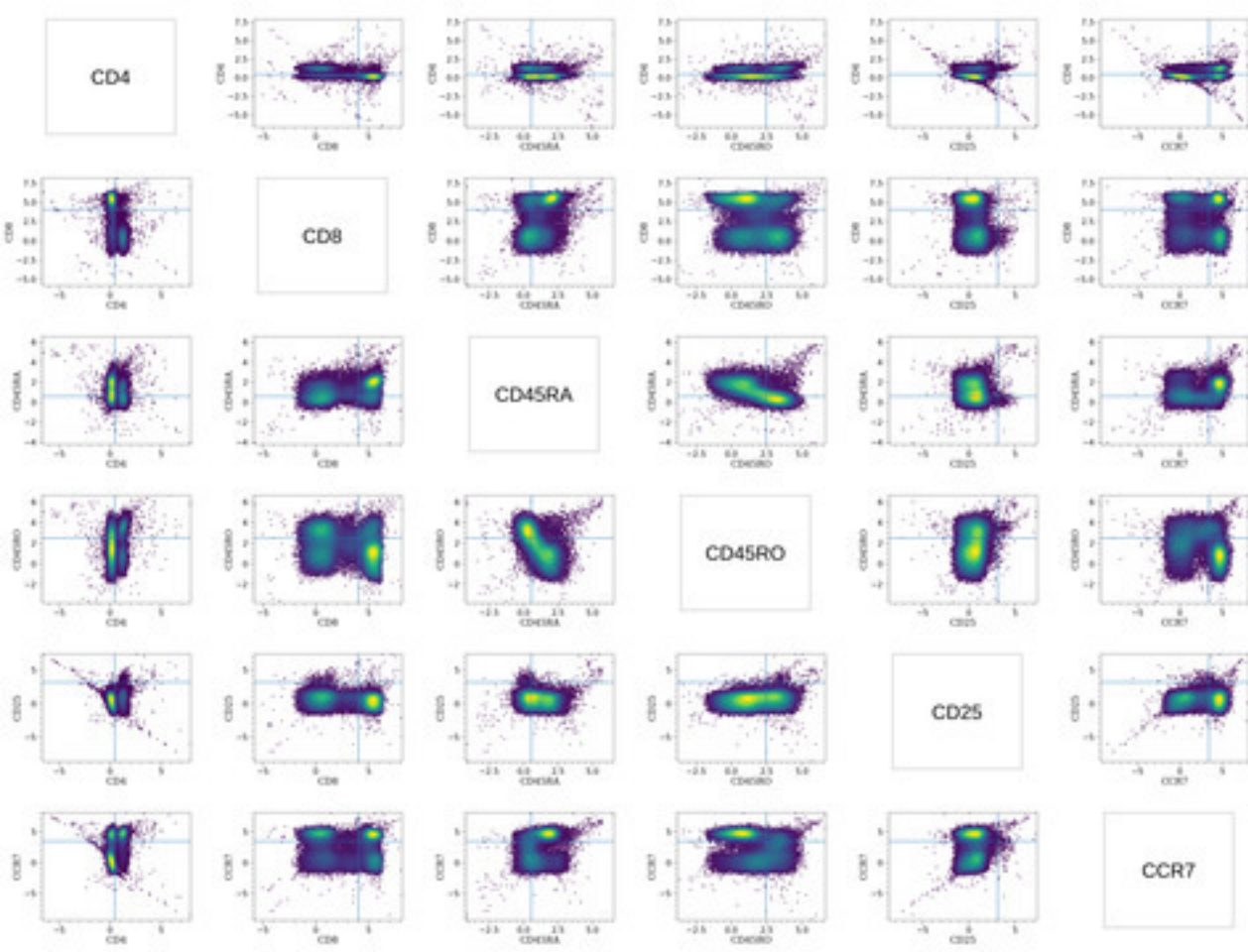

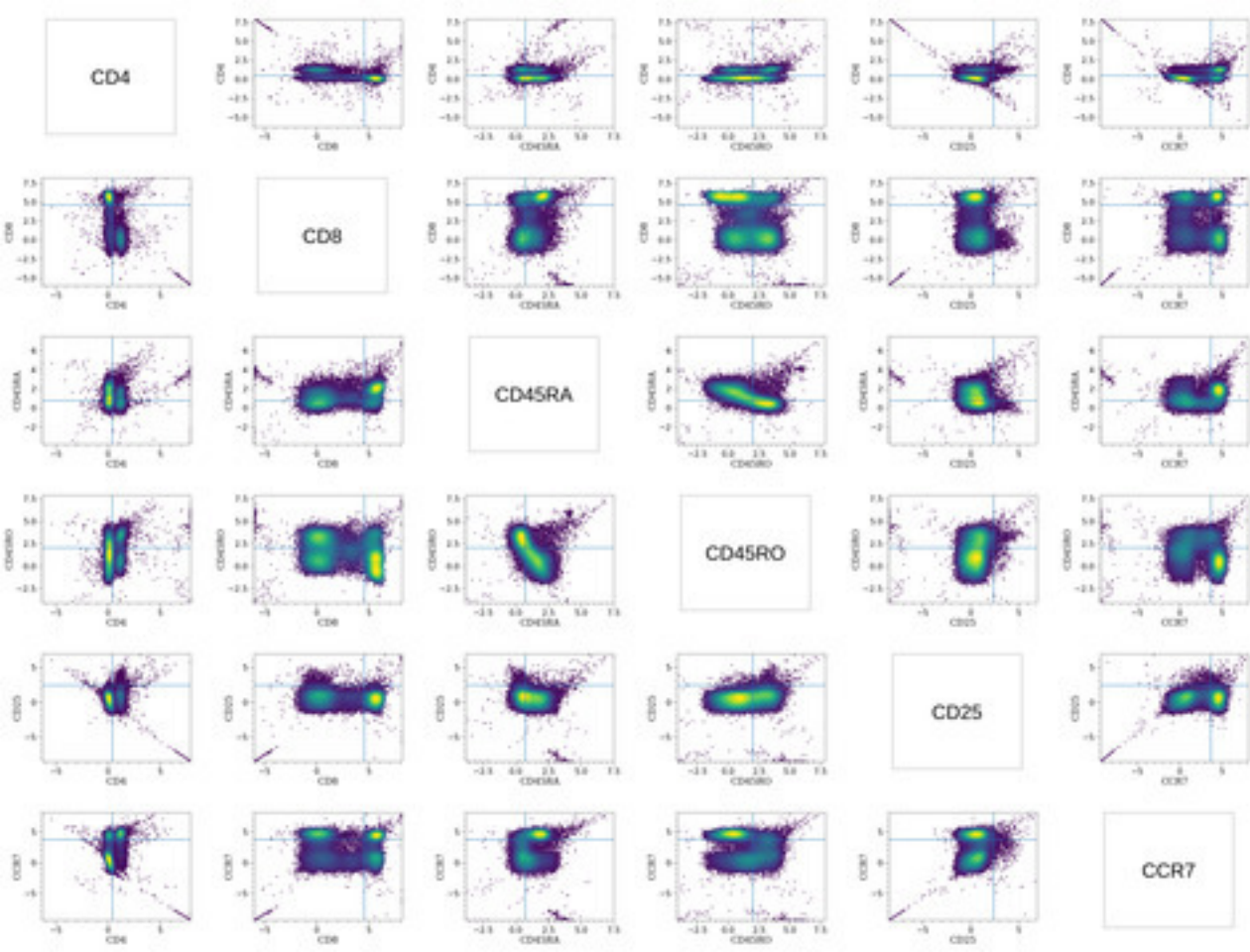

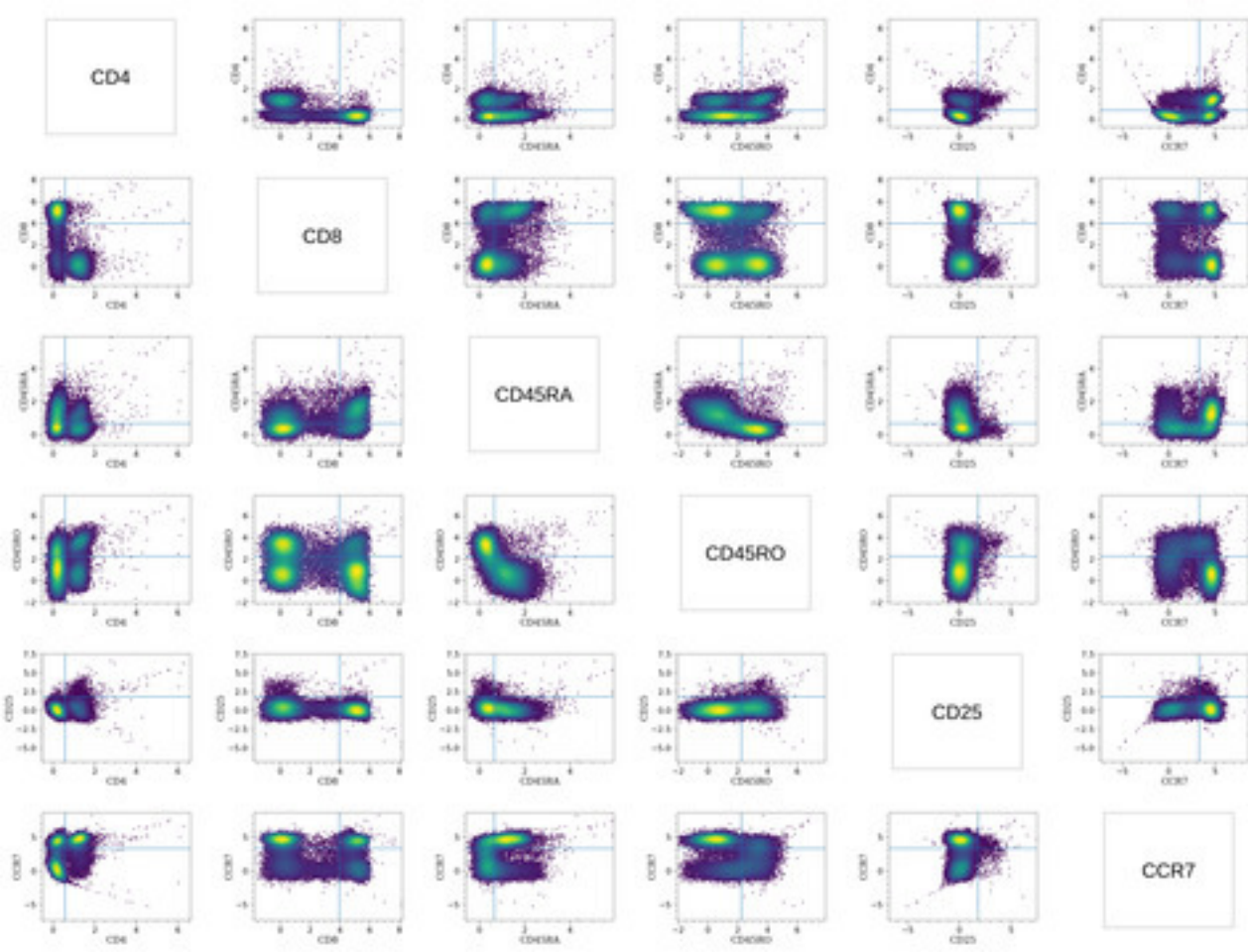

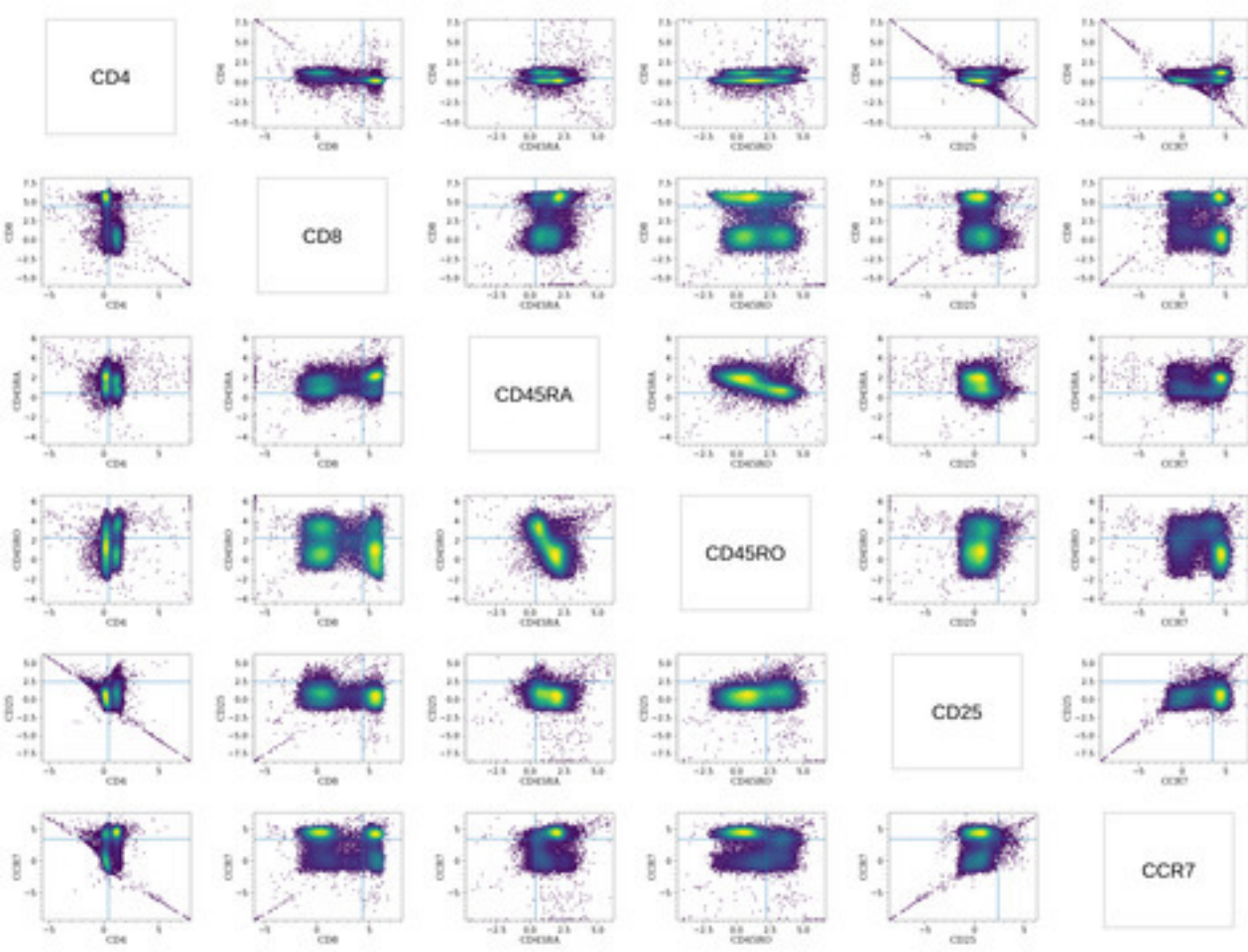

Supplement: Supplementary file 21 — File S4 [file 10038_2020_874_MOESM21_ESM.pdf]

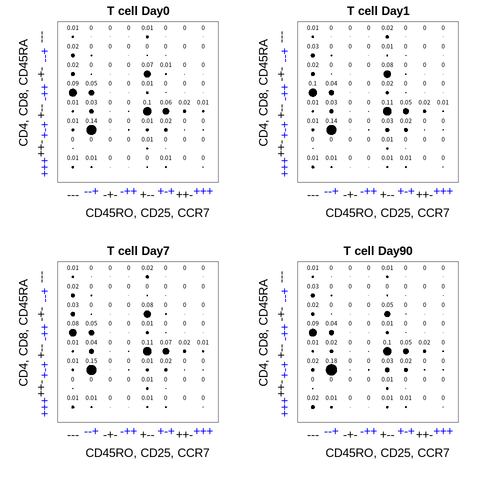

Supplement: Supplementary file 25 — Figure S3 [file 10038_2020_874_MOESM25_ESM.png]

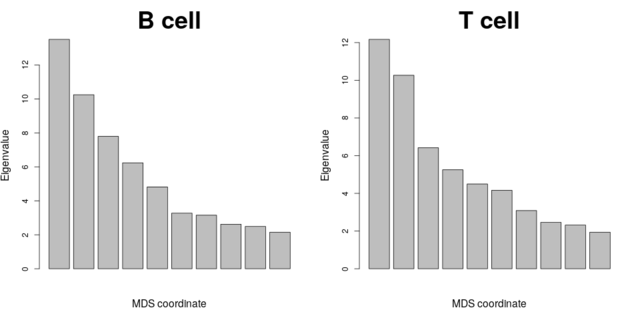

Supplement: Supplementary file 26 — Figure S4 [file 10038_2020_874_MOESM26_ESM.png]

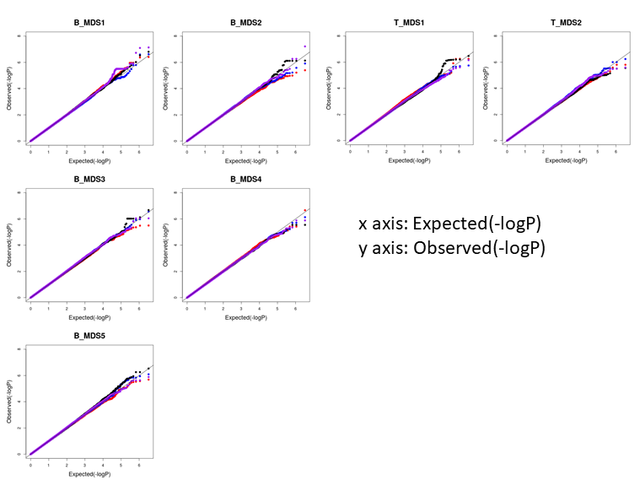

Supplement: Supplementary file 27 — Figure S6 [file 10038_2020_874_MOESM27_ESM.png]
