# Supplementary material for: Genome-wide association study of individual differences of human lymphocyte profiles using large-scale cytometry data
Source: J Hum Genet. 2020 Nov 23;66(6):557–67. doi: 10.1038/s10038-020-00874-x (PMC8144016; doi:10.1038/s10038-020-00874-x)
Supplement: Supplementary file 19 — File S2 [file 10038_2020_874_MOESM19_ESM.pdf]

Method of automatic quantification of lymphocyte subset

The algorithm for discrimination is shown below. First, for each marker of each FACS data, by fitting a mixture normal distribution using the EM algorithm, a cut-off value for determining positive/negative was calculated. We excluded IgM and CD138 in the B cell FACS dataset from this analysis, and all remaining markers showed a bimodal distribution. CD3 in the T cell FACS dataset was also excluded because we had selected CD3-positive cells in the lymphocyte gating process. First, we calculated for one sample (Person ID 1, Day 0) where the number of max iterations was set 10,000. Then, using the result as the initial values of the mean, standard deviations, and mixture ratios, we calculated for all samples where the number of max iterations was set 100,000. We used the normalmixEM function in the R package "mixtools" (ref. 1).

However, depending on the shape of the marker expression value distribution and the presence of outliers, the EM algorithm may not be able to estimate the cut-off value and an extreme value may be estimated. Therefore, to correct such values, we fit a normal distribution to the distribution of the estimated cut-off values of all samples for each marker, and the cut-off values not included in the range between 1st and 99th percentile confidence interval of this normal distribution. Correction was performed by replacing these values with the average value of the fitted normal distribution. In the case of CD25 in T cell FACS, we fitted mixture normal distributions to the distribution of cut-off values and used the right normal distribution for this step. We uploaded the R code of our automatic cell subset quantification process to Github (https://github.com/DaigoOkada/AutoGate2020).

[1] Benaglia T, Chauveau D, Hunter D, Young D. mixtools: An R package for analyzing finite mixture models. 2009;
