# Supplementary material for: Genome-wide association study of individual differences of human lymphocyte profiles using large-scale cytometry data
Source: J Hum Genet. 2020 Nov 23;66(6):557–67. doi: 10.1038/s10038-020-00874-x (PMC8144016; doi:10.1038/s10038-020-00874-x)
Supplement: Supplementary file 22 — File S5 [file 10038_2020_874_MOESM22_ESM.pdf]

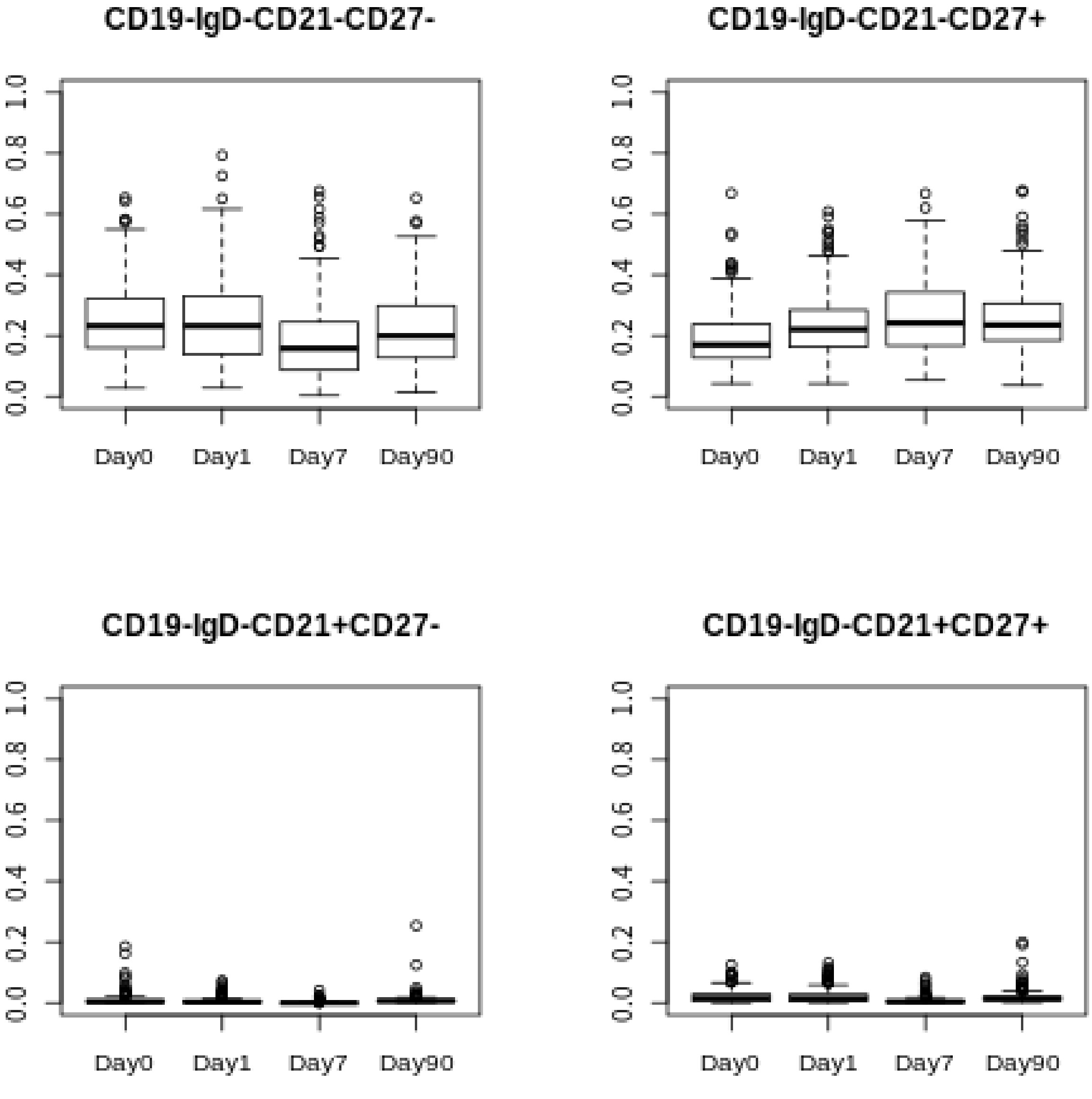
CD19-IgD-CD21-CD27-
CD19-IgD-CD21-CD27+
CD19-IgD-CD21+CD27-
CD19-IgD-CD21+CD27+
0.0
0.2
0.4
0.6
0.8
1.0
Day0
Day1
Day7
Day90

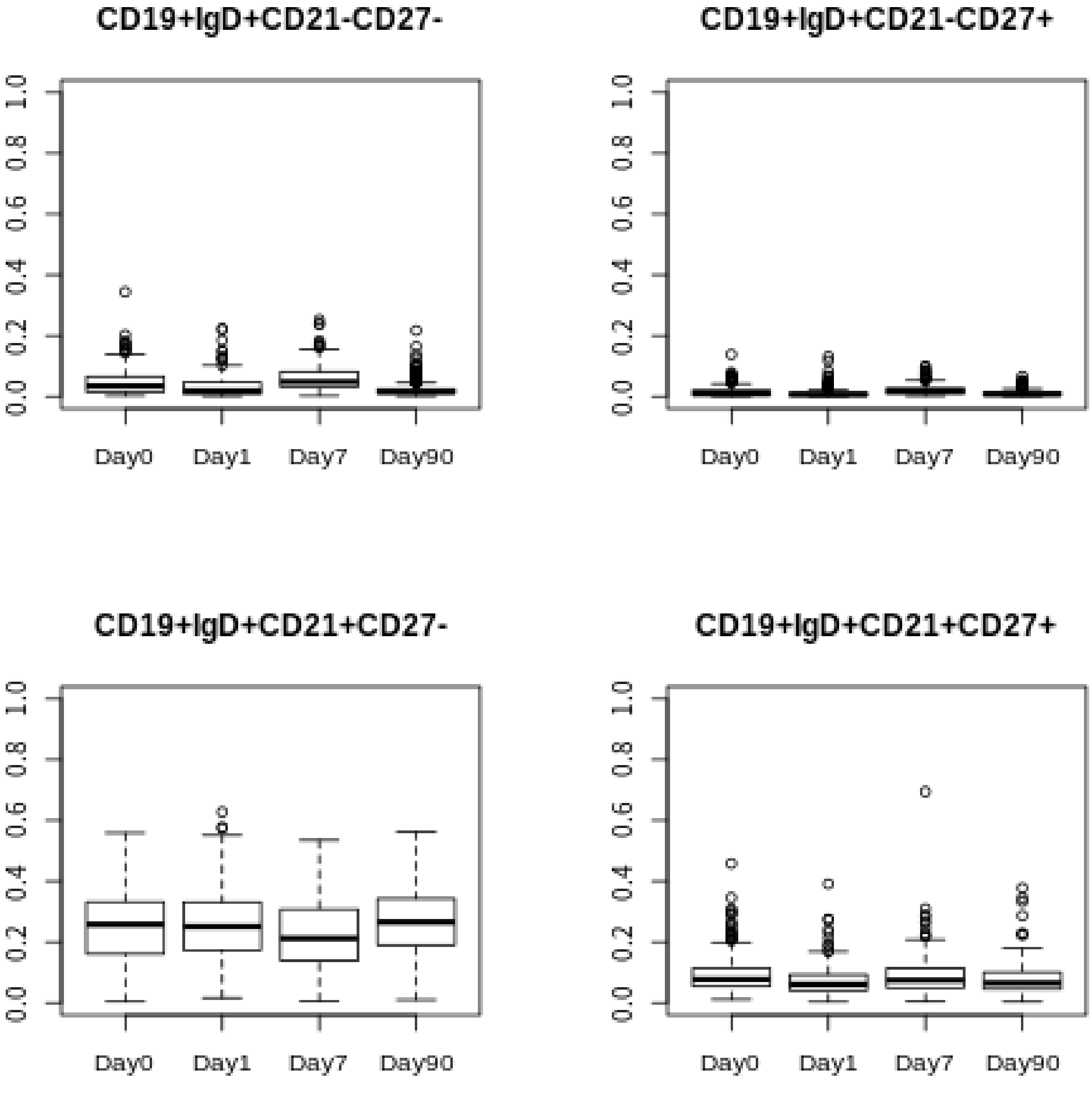
CD19+IgD+CD21-CD27-
CD19+IgD+CD21-CD27+
CD19+IgD+CD21+CD27-
CD19+IgD+CD21+CD27+
0.0
0.2
0.4
0.6
0.8
1.0
Day0
Day1
Day7
Day90

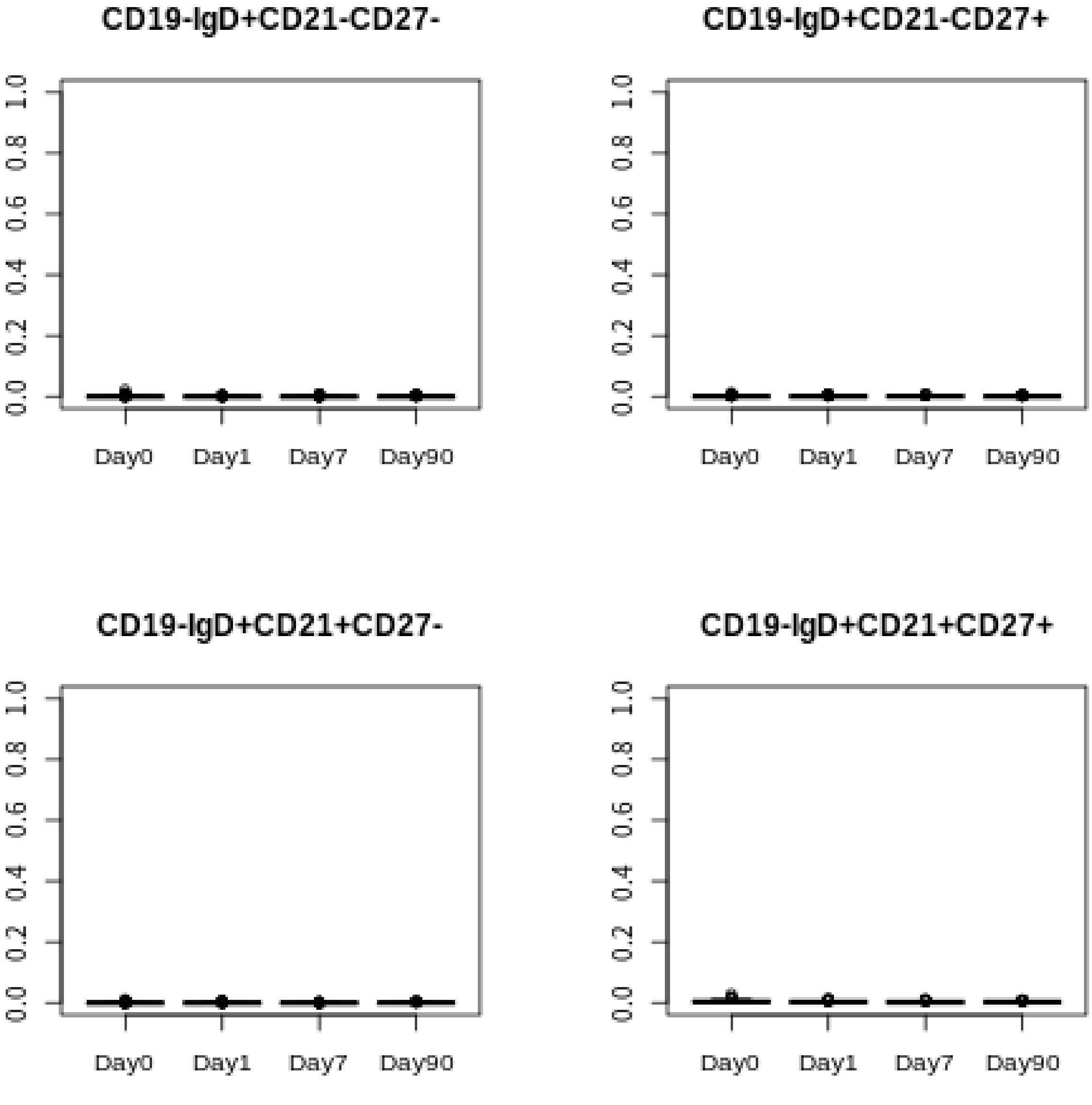
CD19-IgD+CD21-CD27-
CD19-IgD+CD21-CD27+
CD19-IgD+CD21+CD27-
CD19-IgD+CD21+CD27+
0.0
0.2
0.4
0.6
0.8
1.0
Day0
Day1
Day7
Day90

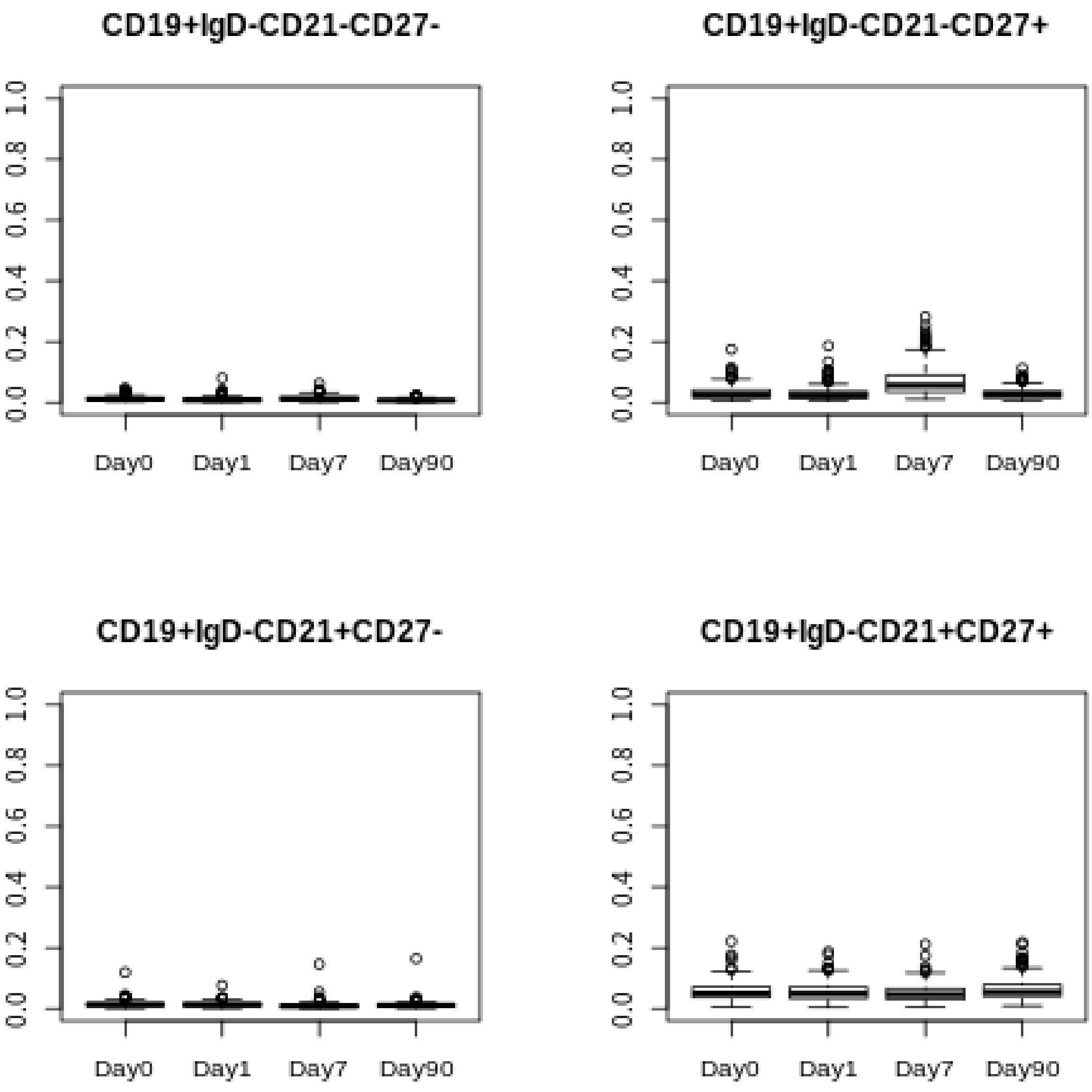

CD19+IgD-CD21-CD27-
CD19+IgD-CD21-CD27+
CD19+IgD-CD21+CD27-
CD19+IgD-CD21+CD27+
0.0
0.2
0.4
0.6
0.8
1.0
Day0
Day1
Day7
Day90
