# Supplementary material for: Genome-wide association study of individual differences of human lymphocyte profiles using large-scale cytometry data
Source: J Hum Genet. 2020 Nov 23;66(6):557–67. doi: 10.1038/s10038-020-00874-x (PMC8144016; doi:10.1038/s10038-020-00874-x)
Supplement: Supplementary file 23 — File S7 [file 10038_2020_874_MOESM23_ESM.pdf]

Interpretation of MDS coordinates

B_MDS1 showed no significant association with any subset. This coordinate is thought to correspond to complex changes that do not depend on a particular subset. B_MDS2 showed a significant negative association with CD19+IgD+CD21-CD27+ and CD19+IgD+CD21+CD27+. CD19+IgD+CD21-CD27+ contains plasma cells and CD19+IgD+CD21+CD27+ is annotated to non-switched memory B cells. B_MDS3 showed a significant negative association with CD19+IgD+CD21+CD27-, which is annotated to naive B cells. B_MDS4 and B_MDS5 also showed a significant negative association with CD19-IgD-CD21-CD27+, which contains plasma cells.

T_MDS1 showed a negative correlation with CD4-CD8-CD45RA-CD45RO+CD25-CCR7+ and CD4-CD8+CD45RA-CD45RO+CD25-CCR7-, which are annotated to CD4-CD8- memory T cells and CD8+ memory T cells, respectively. In addition, T_MDS1 showed a positive correlation with CD4+CD8-CD45RA+CD45RO-CD25-CCR7+ and CD4+CD8-CD45RA+CD45RO-CD25+CCR7-, which are annotated to CD4+ naive T cells and resting Tregs, respectively.

T_MDS2 showed a negative correlation with CD4-CD8+CD45RA+CD45RO-CD25-CCR7+ and CD4+CD8-CD45RA+CD45RO-CD25-CCR7+, which are annotated to CD8+ naive T cells and CD4+ naive T cells, respectively. In addition, T_MDS2 showed a positive correlation with CD4-CD8+CD45RA+CD45RO-CD25-CCR7-, which is annotated to CD8+ exhausted T cells.
