# Supplementary material for: Genome-wide association study of individual differences of human lymphocyte profiles using large-scale cytometry data
Source: J Hum Genet. 2020 Nov 23;66(6):557–67. doi: 10.1038/s10038-020-00874-x (PMC8144016; doi:10.1038/s10038-020-00874-x)
Supplement: Supplementary file 24 — File S6 [file 10038_2020_874_MOESM24_ESM.pdf]

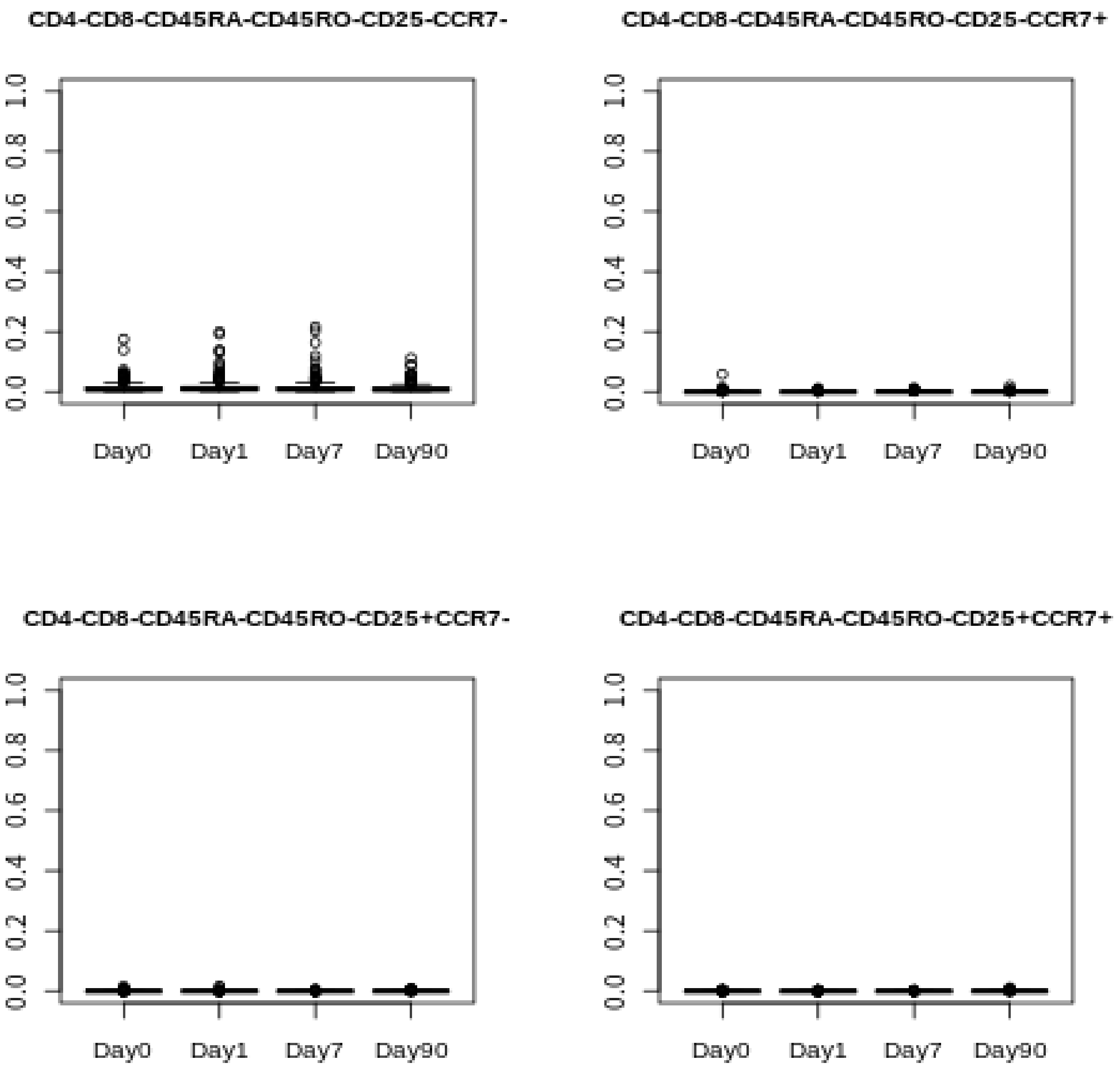
CD4-CD8-CD45RA-CD45RO-CD25-CCR7-
CD4-CD8-CD45RA-CD45RO-CD25-CCR7+
CD4-CD8-CD45RA-CD45RO-CD25+CCR7-
CD4-CD8-CD45RA-CD45RO-CD25+CCR7+
0.0
0.2
0.4
0.6
0.8
1.0
Day0
Day1
Day7
Day90

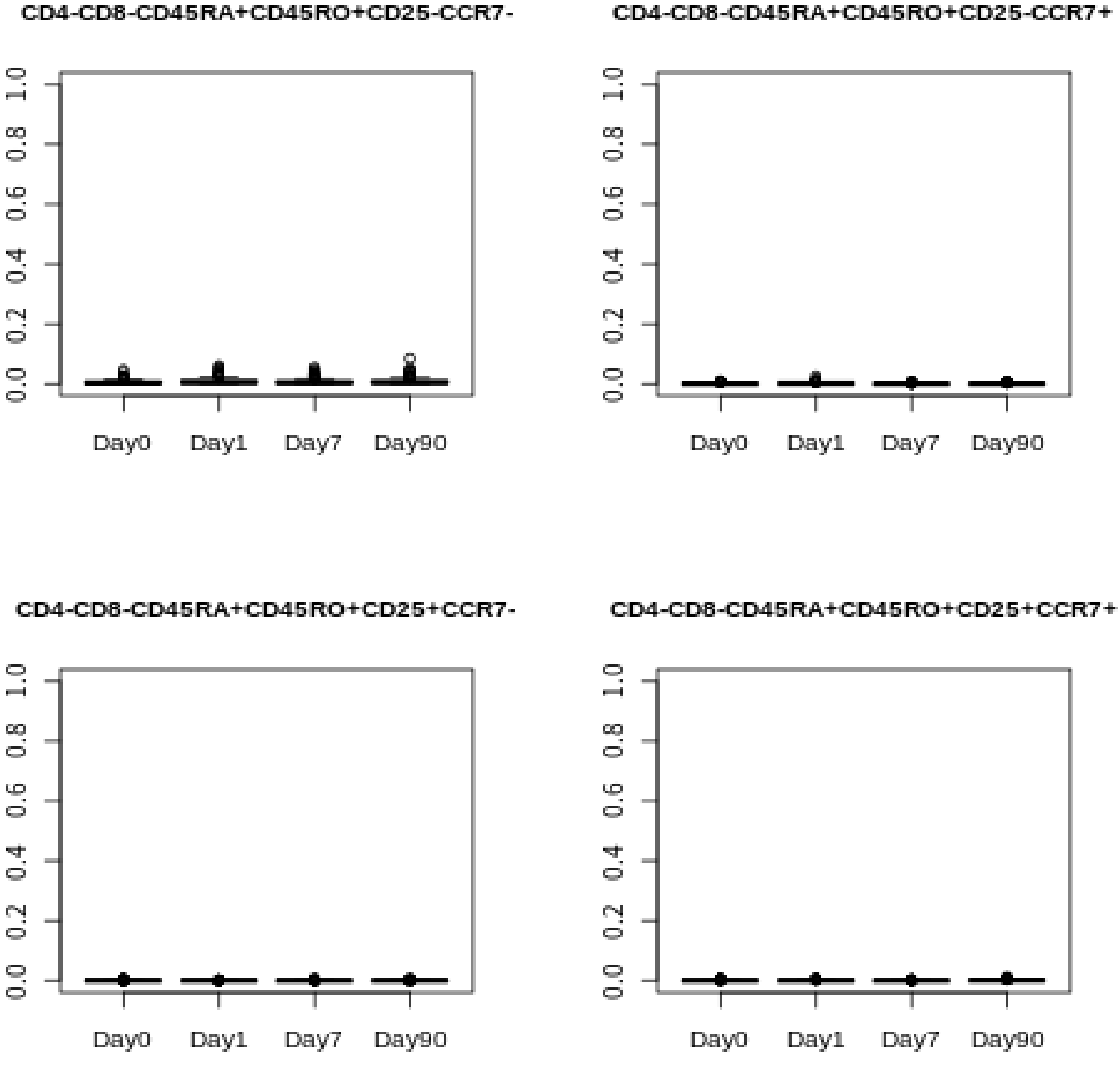
CD4-CD8-CD45RA+CD45RO+CD25-CCR7-
CD4-CD8-CD45RA+CD45RO+CD25-CCR7+
CD4-CD8-CD45RA+CD45RO+CD25+CCR7-
CD4-CD8-CD45RA+CD45RO+CD25+CCR7+
0.0
0.2
0.4
0.6
0.8
1.0
Day0
Day1
Day7
Day90

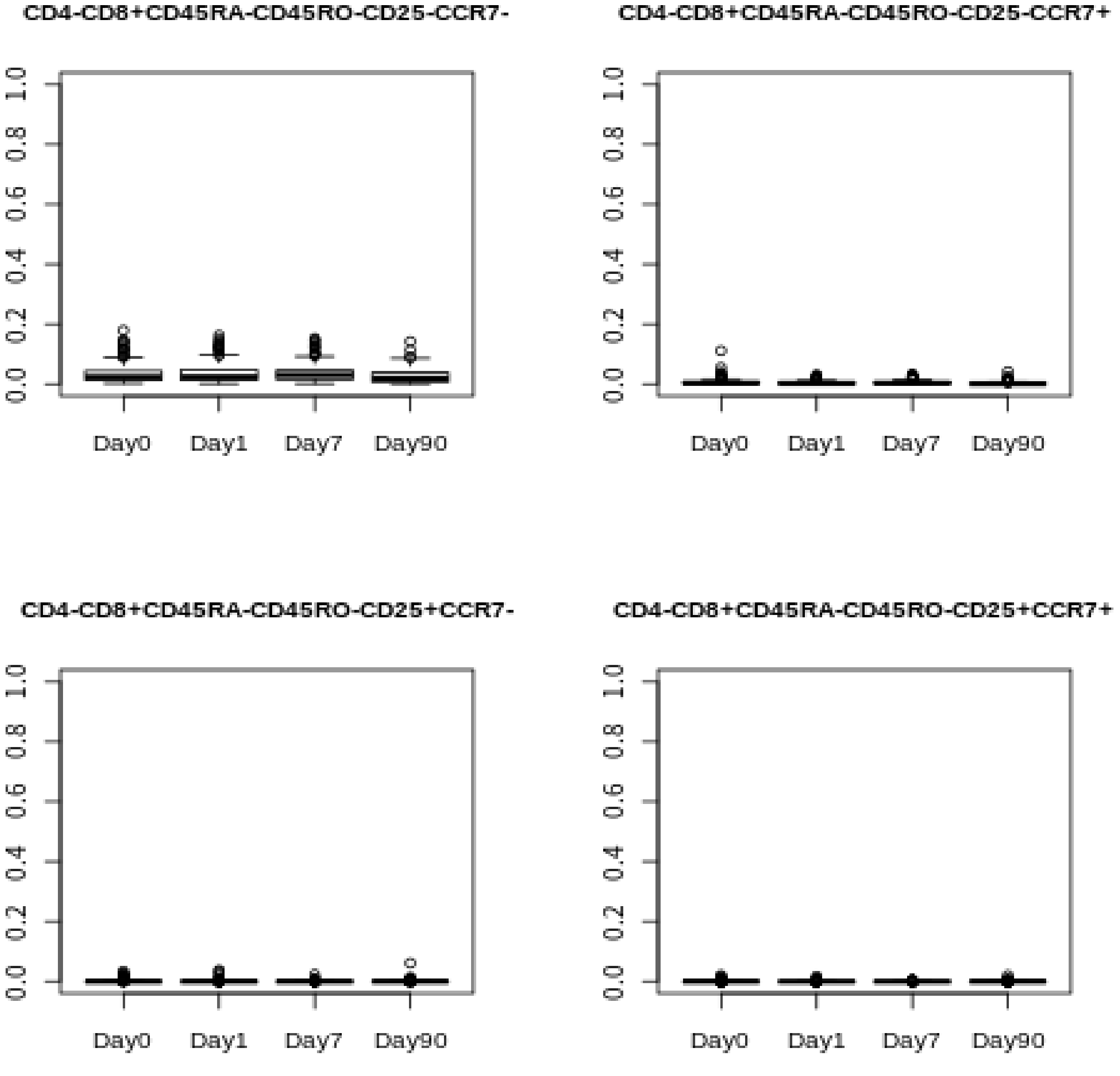
CD4-CD8+CD45RA-CD45RO-CD25-CCR7-
CD4-CD8+CD45RA-CD45RO-CD25-CCR7+
CD4-CD8+CD45RA-CD45RO-CD25+CCR7-
CD4-CD8+CD45RA-CD45RO-CD25+CCR7+
0.0
0.2
0.4
0.6
0.8
1.0
Day0
Day1
Day7
Day90

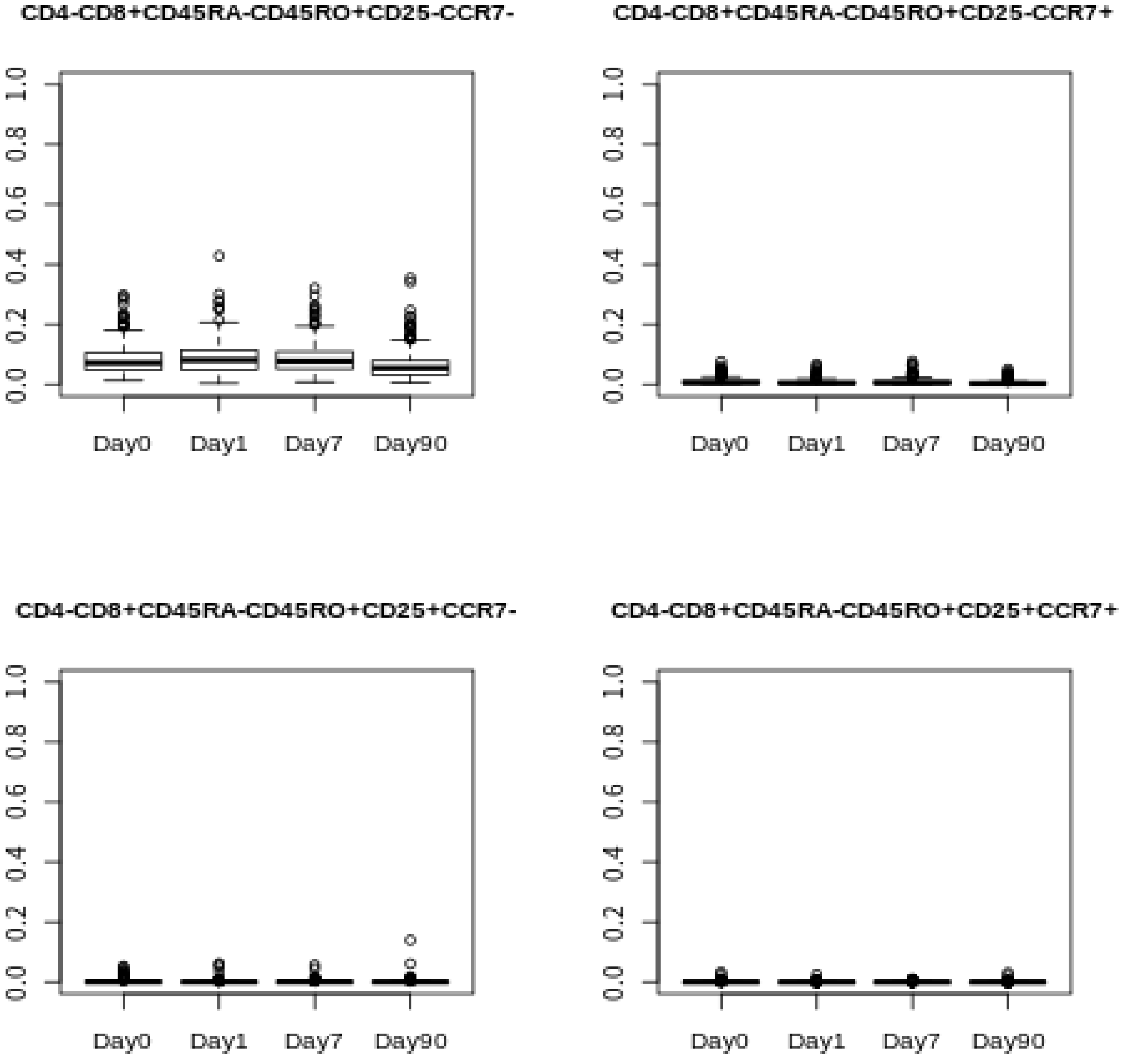
CD4-CD8+CD45RA-CD45RO+CD25-CCR7-
CD4-CD8+CD45RA-CD45RO+CD25-CCR7+
CD4-CD8+CD45RA-CD45RO+CD25+CCR7-
CD4-CD8+CD45RA-CD45RO+CD25+CCR7+
0.0
0.2
0.4
0.6
0.8
1.0
Day0
Day1
Day7
Day90

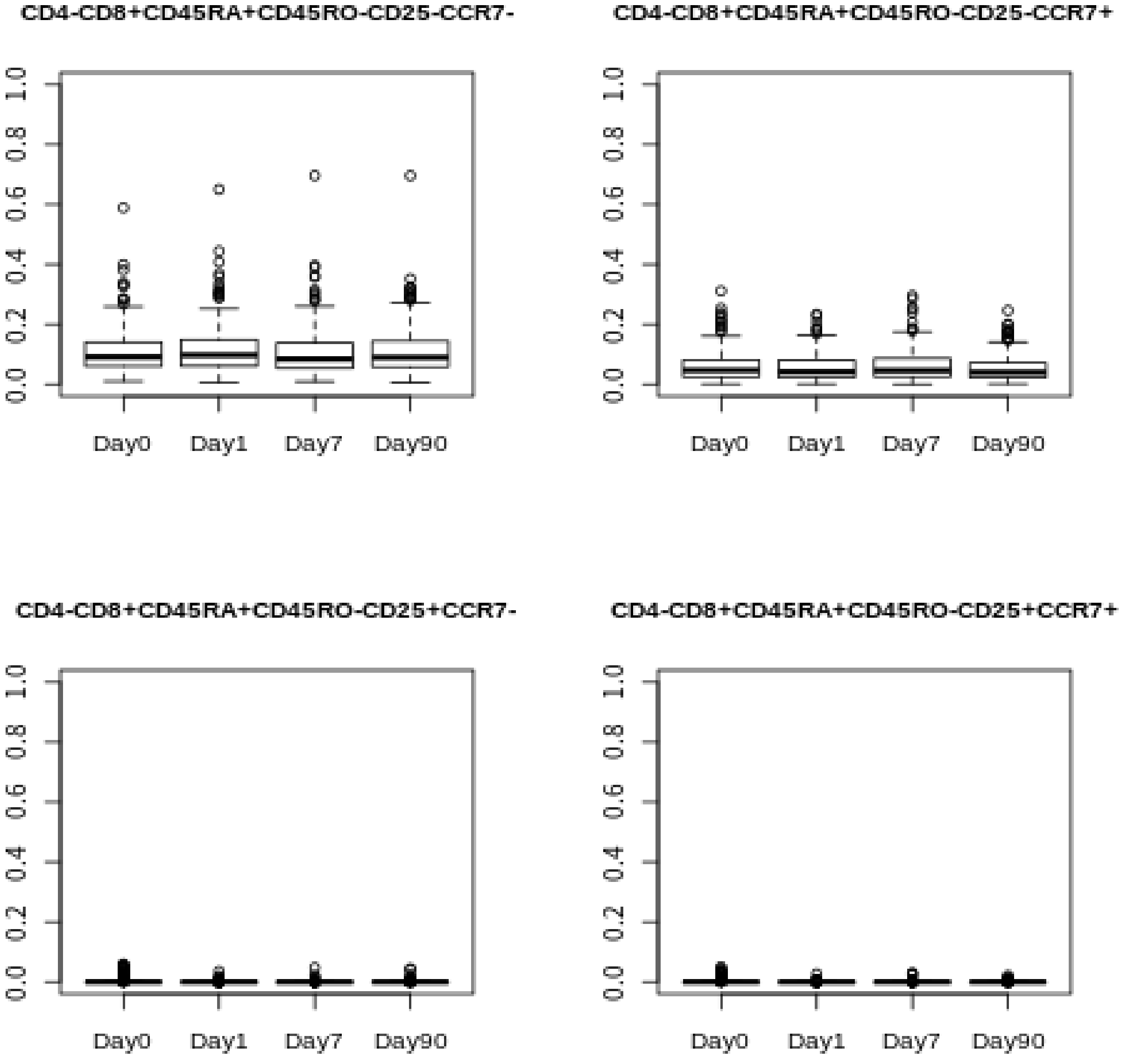
CD4-CD8+CD45RA+CD45RO-CD25-CCR7-
CD4-CD8+CD45RA+CD45RO-CD25-CCR7+
CD4-CD8+CD45RA+CD45RO-CD25+CCR7-
CD4-CD8+CD45RA+CD45RO-CD25+CCR7+
0.0
0.2
0.4
0.6
0.8
1.0
Day0
Day1
Day7
Day90

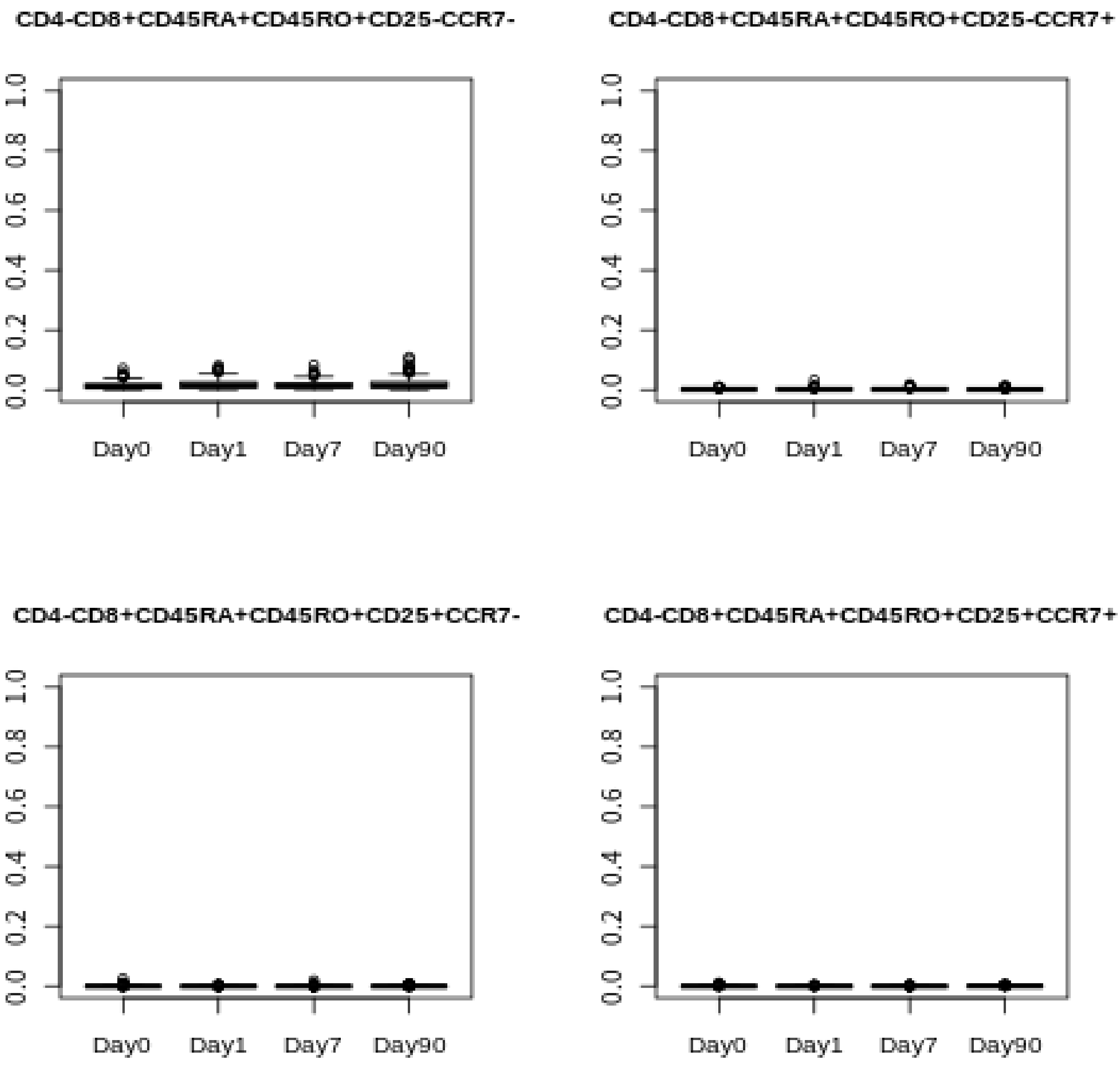
CD4-CD8+CD45RA+CD45RO+CD25-CCR7-
CD4-CD8+CD45RA+CD45RO+CD25-CCR7+
CD4-CD8+CD45RA+CD45RO+CD25+CCR7-
CD4-CD8+CD45RA+CD45RO+CD25+CCR7+
0.0
0.2
0.4
0.6
0.8
1.0
Day0
Day1
Day7
Day90

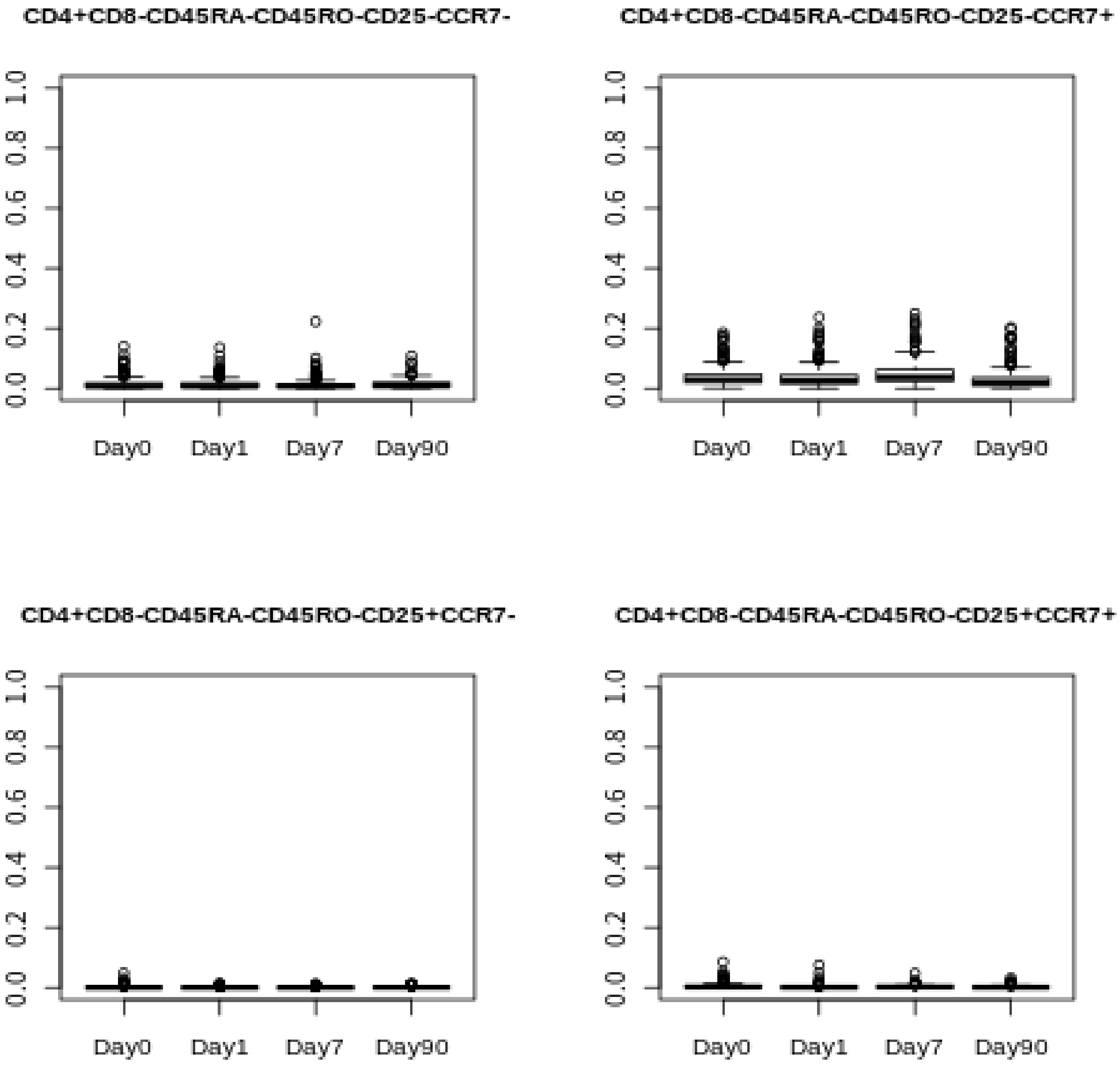
CD4+CD8-CD45RA-CD45RO-CD25-CCR7-
CD4+CD8-CD45RA-CD45RO-CD25-CCR7+
CD4+CD8-CD45RA-CD45RO-CD25+CCR7-
CD4+CD8-CD45RA-CD45RO-CD25+CCR7+
0.0 0.2 0.4 0.6 0.8 1.0
Day0 Day1 Day7 Day90

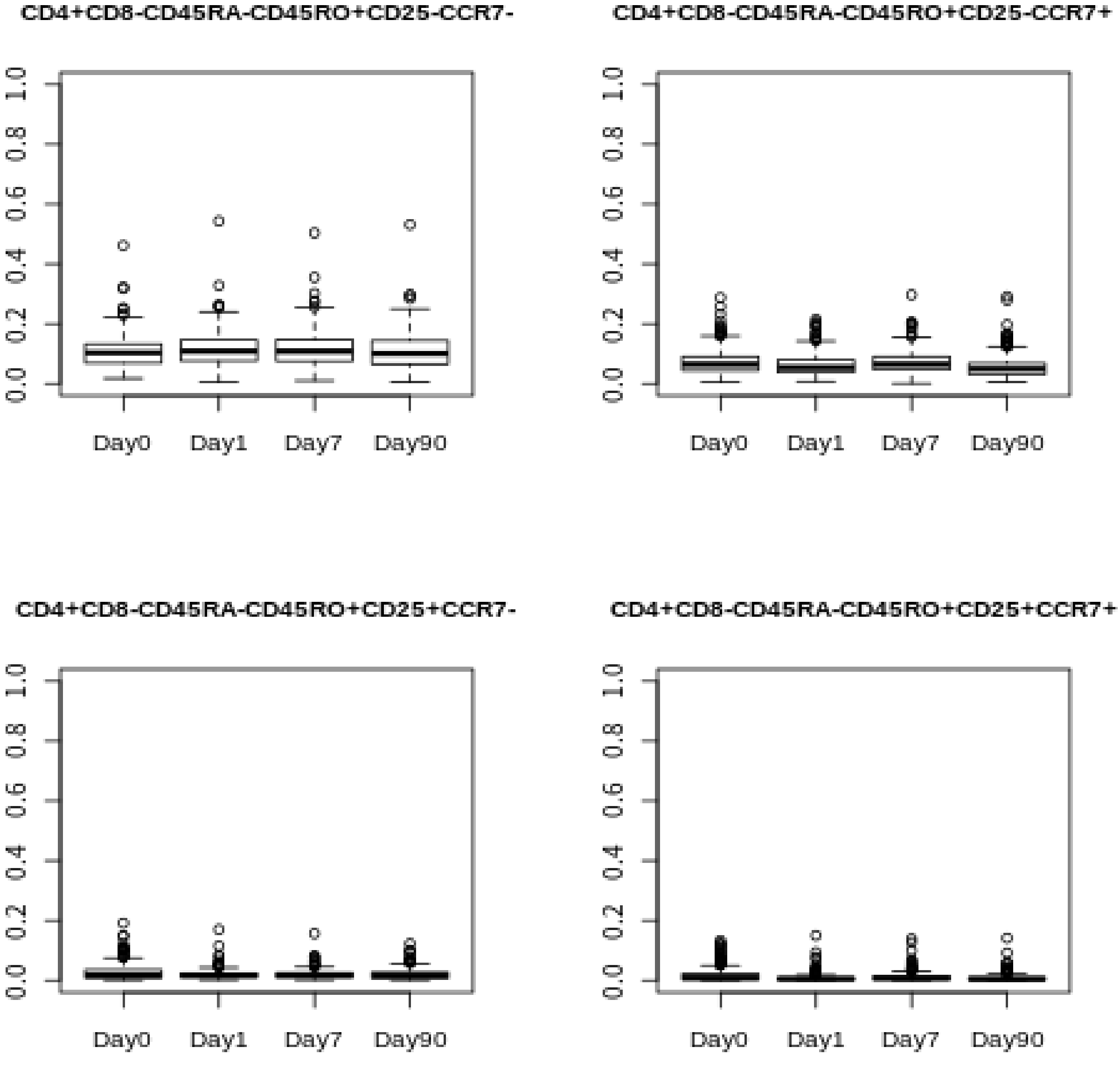
CD4+CD8-CD45RA-CD45RO+CD25-CCR7-
CD4+CD8-CD45RA-CD45RO+CD25-CCR7+
CD4+CD8-CD45RA-CD45RO+CD25+CCR7-
CD4+CD8-CD45RA-CD45RO+CD25+CCR7+
0.0
0.2
0.4
0.6
0.8
1.0
Day0
Day1
Day7
Day90

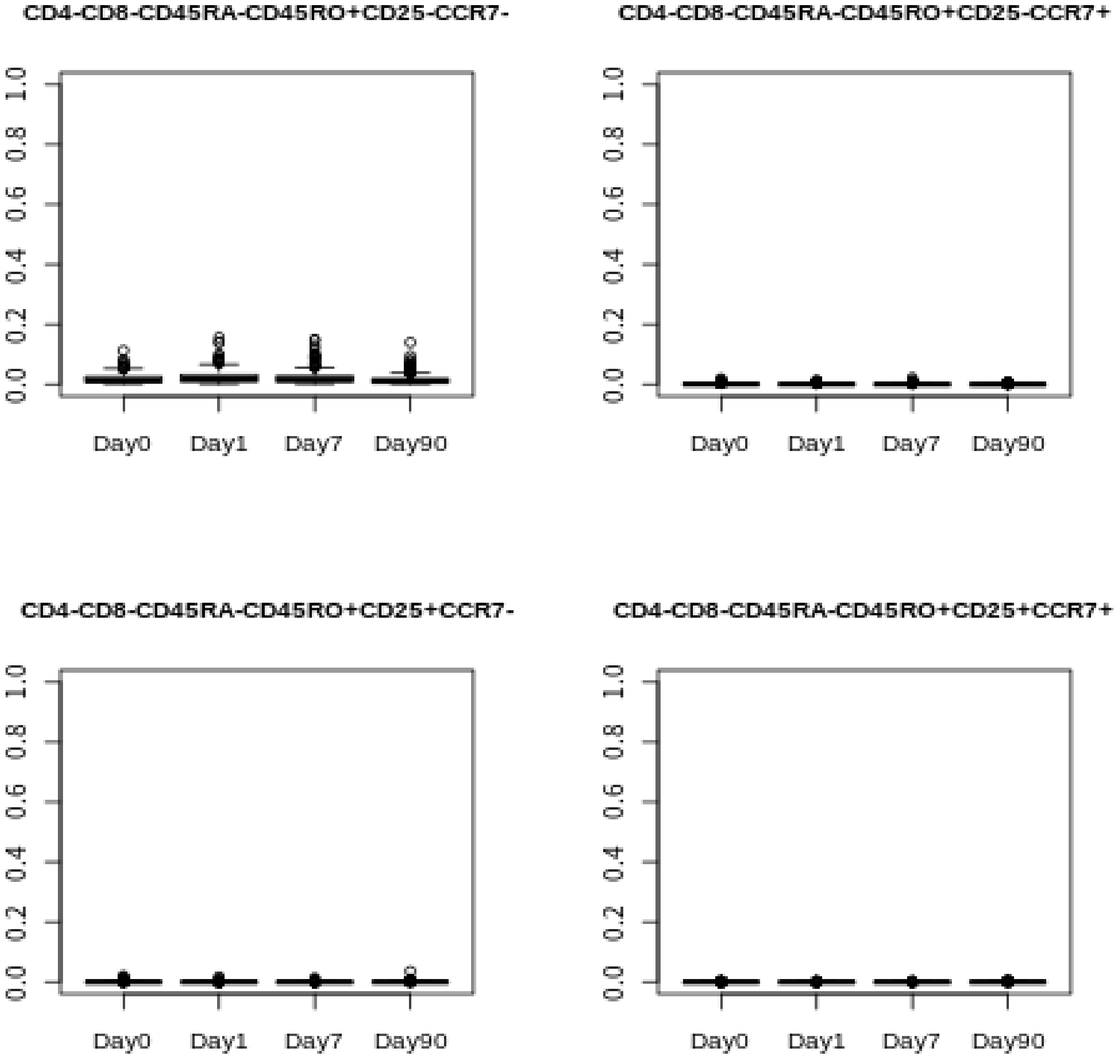
CD4-CD8-CD45RA-CD45RO+CD25-CCR7-
CD4-CD8-CD45RA-CD45RO+CD25-CCR7+
CD4-CD8-CD45RA-CD45RO+CD25+CCR7-
CD4-CD8-CD45RA-CD45RO+CD25+CCR7+
0.0
0.2
0.4
0.6
0.8
1.0
Day0
Day1
Day7
Day90

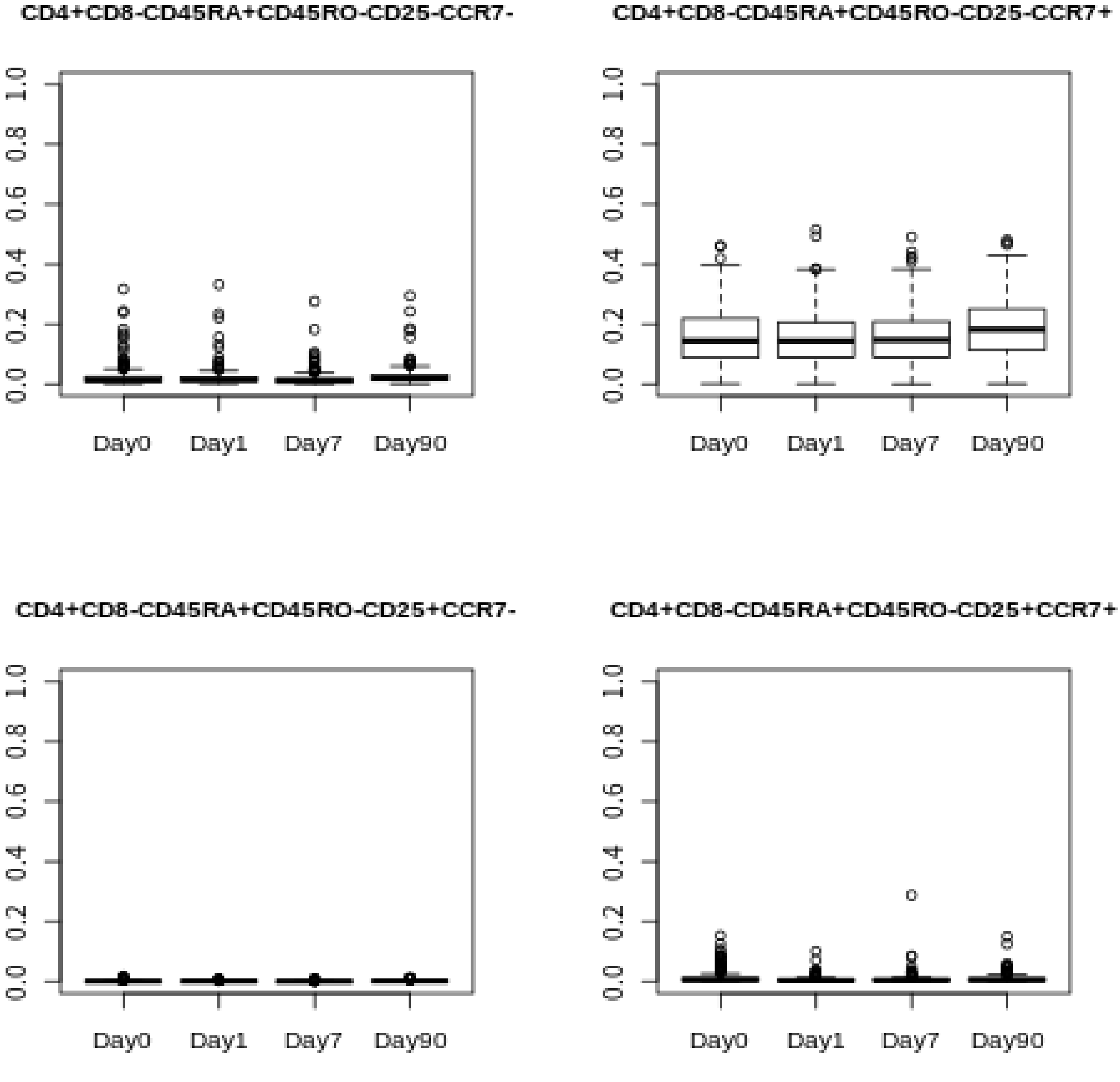
CD4+CD8-CD45RA+CD45RO-CD25-CCR7-
CD4+CD8-CD45RA+CD45RO-CD25-CCR7+
CD4+CD8-CD45RA+CD45RO-CD25+CCR7-
CD4+CD8-CD45RA+CD45RO-CD25+CCR7+
0.0
0.2
0.4
0.6
0.8
1.0
Day0
Day1
Day7
Day90

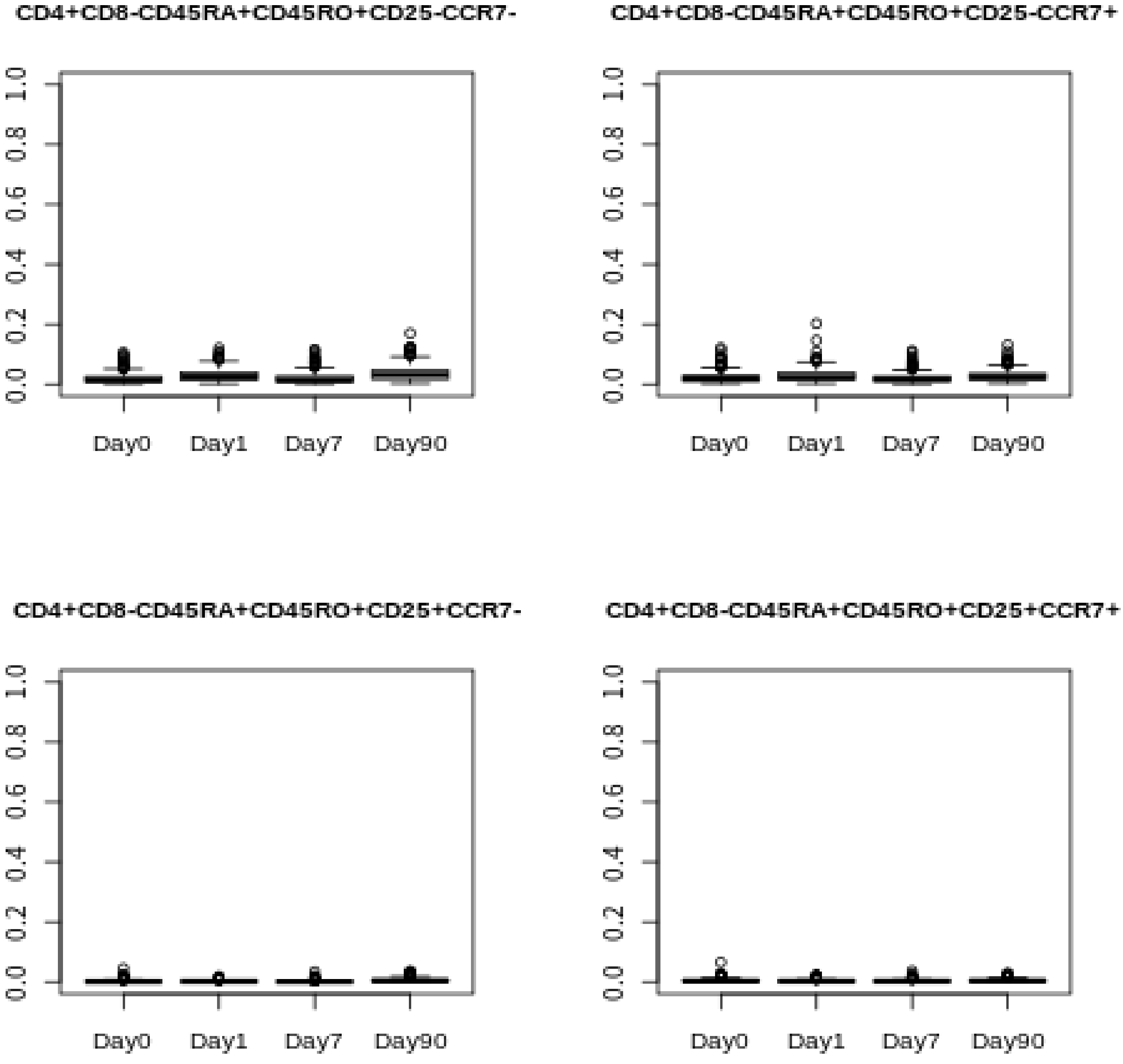
CD4+CD8-CD45RA+CD45RO+CD25-CCR7-
CD4+CD8-CD45RA+CD45RO+CD25-CCR7+
CD4+CD8-CD45RA+CD45RO+CD25+CCR7-
CD4+CD8-CD45RA+CD45RO+CD25+CCR7+
0.0
0.2
0.4
0.6
0.8
1.0
Day0
Day1
Day7
Day90

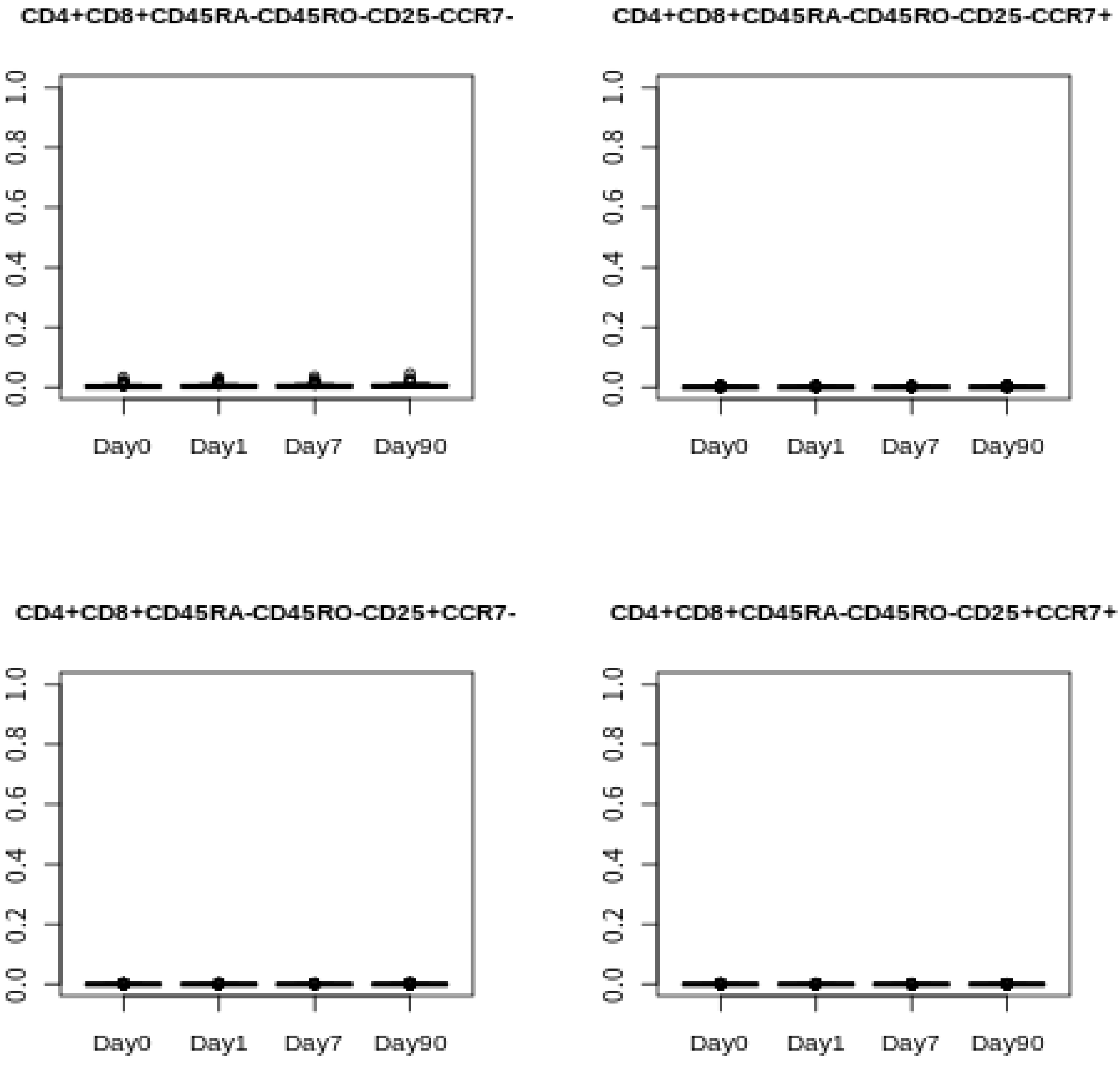
CD4+CD8+CD45RA-CD45RO-CD25-CCR7-
CD4+CD8+CD45RA-CD45RO-CD25-CCR7+
CD4+CD8+CD45RA-CD45RO-CD25+CCR7-
CD4+CD8+CD45RA-CD45RO-CD25+CCR7+
0.0
0.2
0.4
0.6
0.8
1.0
Day0
Day1
Day7
Day90

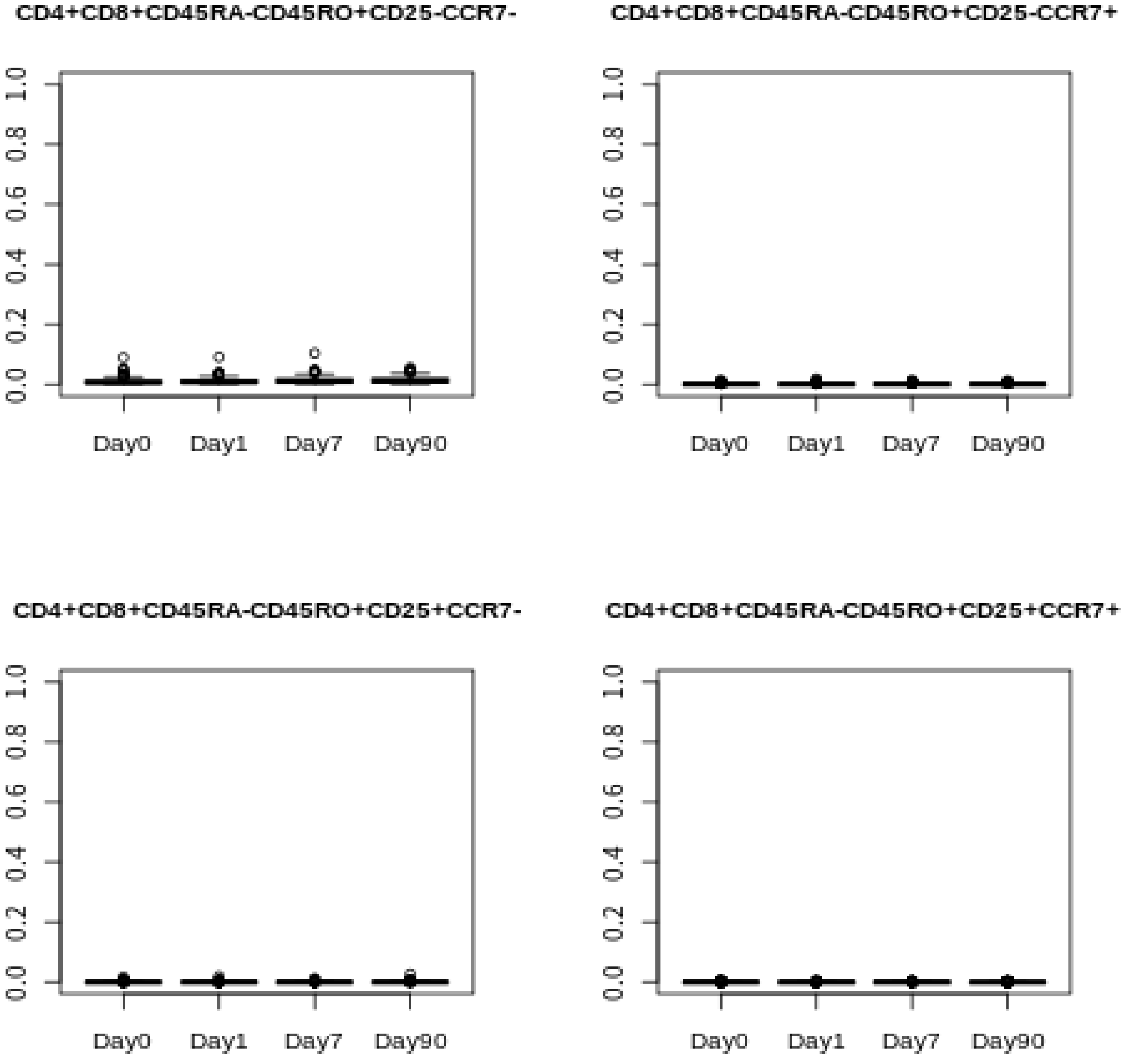
CD4+CD8+CD45RA-CD45RO+CD25-CCR7-
CD4+CD8+CD45RA-CD45RO+CD25-CCR7+
CD4+CD8+CD45RA-CD45RO+CD25+CCR7-
CD4+CD8+CD45RA-CD45RO+CD25+CCR7+
0.0
0.2
0.4
0.6
0.8
1.0
Day0
Day1
Day7
Day90

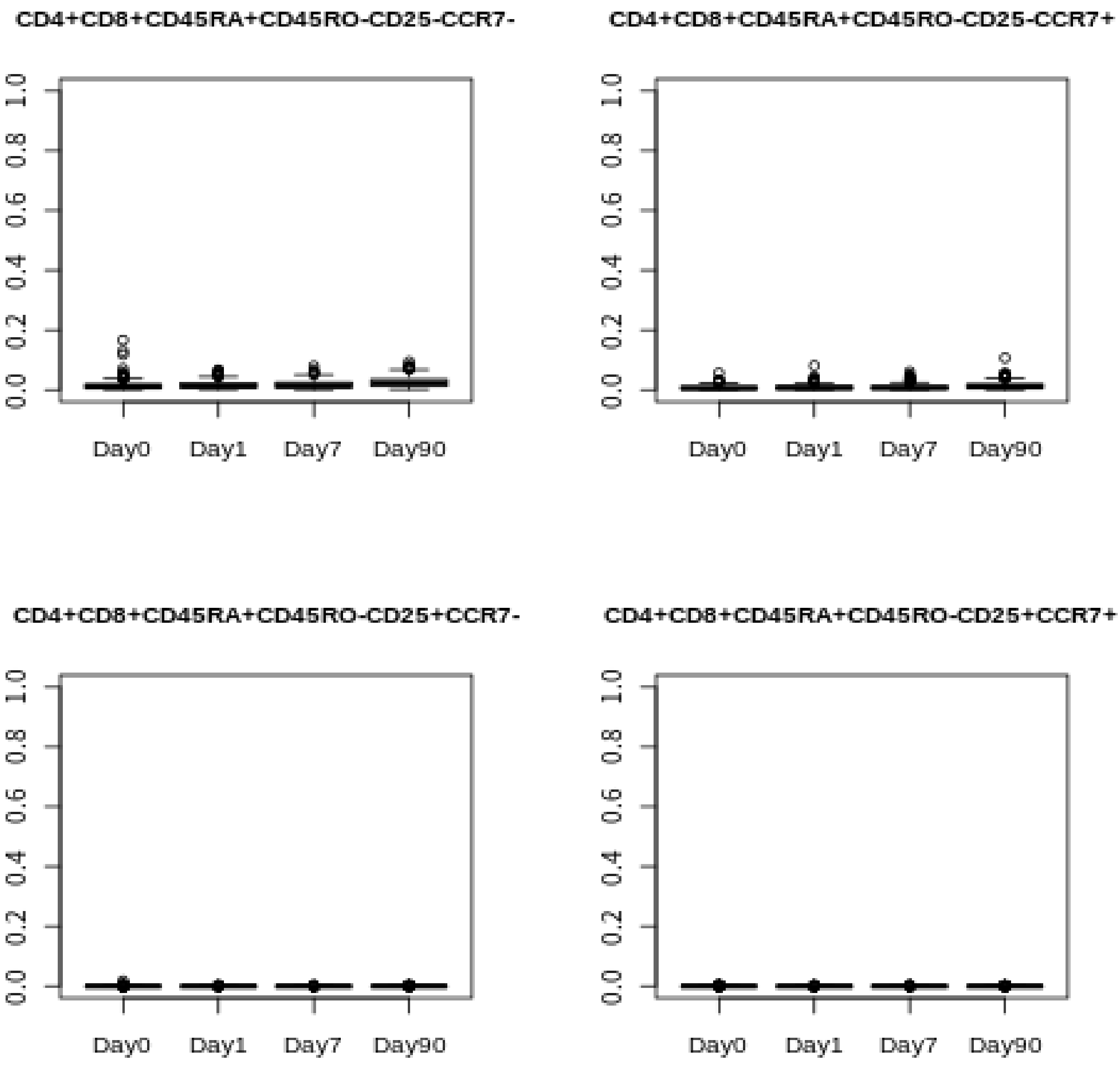
CD4+CD8+CD45RA+CD45RO-CD25-CCR7-
CD4+CD8+CD45RA+CD45RO-CD25-CCR7+
CD4+CD8+CD45RA+CD45RO-CD25+CCR7-
CD4+CD8+CD45RA+CD45RO-CD25+CCR7+
0.0
0.2
0.4
0.6
0.8
1.0
Day0
Day1
Day7
Day90

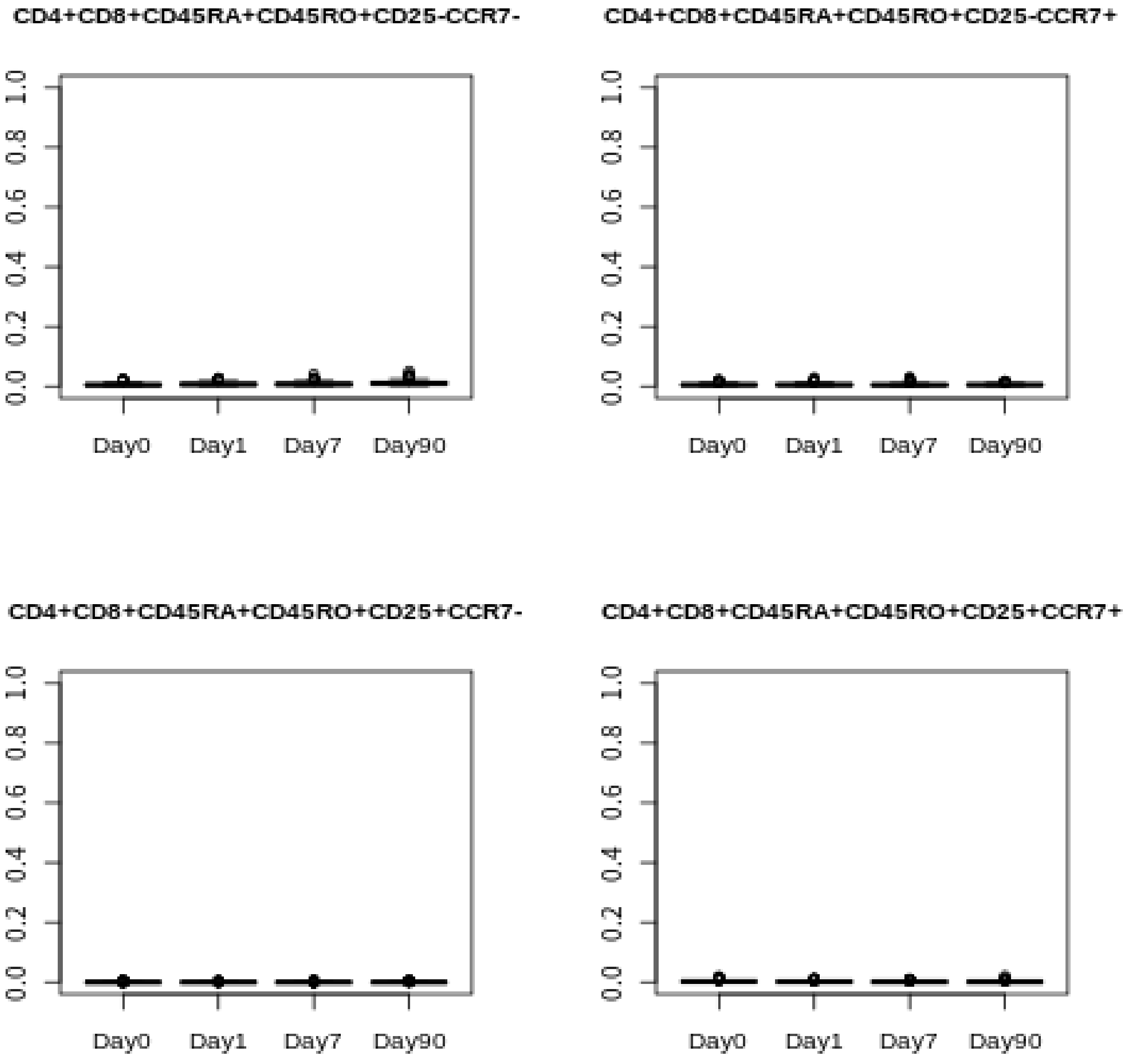
CD4+CD8+CD45RA+CD45RO+CD25-CCR7-
CD4+CD8+CD45RA+CD45RO+CD25-CCR7+
CD4+CD8+CD45RA+CD45RO+CD25+CCR7-
CD4+CD8+CD45RA+CD45RO+CD25+CCR7+
0.0
0.2
0.4
0.6
0.8
1.0
Day0
Day1
Day7
Day90

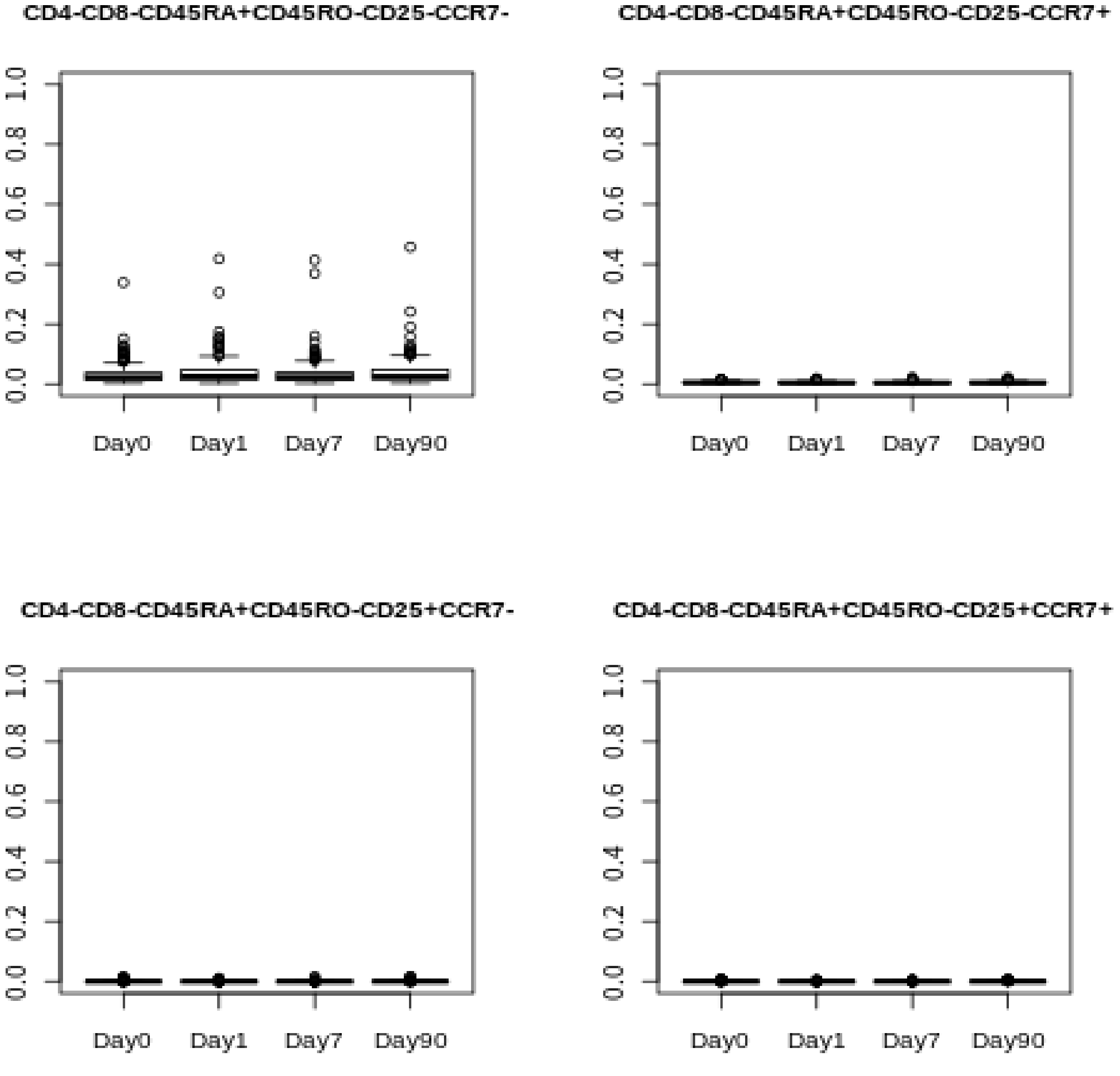
CD4-CD8-CD45RA+CD45RO-CD25-CCR7-
CD4-CD8-CD45RA+CD45RO-CD25-CCR7+
CD4-CD8-CD45RA+CD45RO-CD25+CCR7-
CD4-CD8-CD45RA+CD45RO-CD25+CCR7+
0.0
0.2
0.4
0.6
0.8
1.0
Day0
Day1
Day7
Day90
